# Metformin promotes mitochondrial integrity through AMPK‐signaling in Leber's hereditary optic neuropathy

**DOI:** 10.1002/2211-5463.70165

**Published:** 2025-11-23

**Authors:** Chatnapa Panusatid, Rapasviranda Soiyangsuk, Maneeluck Tanadjindarat, Chayanon Peerapittayamongkol

**Affiliations:** ^1^ Department of Biochemistry, Faculty of Medicine Siriraj Hospital Mahidol University Bangkok Thailand

**Keywords:** AMPK activation, Leber's hereditary optic neuropathy, Metformin, Mitochondrial dynamics, Mitophagy, Primary fibroblasts

## Abstract

Leber's hereditary optic neuropathy (LHON) is a maternally inherited disorder caused by mitochondrial DNA mutations in complex I of the respiratory chain, leading to impaired ATP production, mitochondrial fragmentation, and oxidative stress that contribute to vision loss. This study investigated the potential repurposing of metformin, a widely used antidiabetic drug, in fibroblasts from LHON patients carrying the m.11778G>A mutation. Fibroblasts from LHON patients and healthy individuals were treated with metformin, and mitochondrial function was assessed using high‐content imaging, biochemical assays, immunoblotting, immunofluorescence, and Seahorse analysis. Metformin reduced mitochondrial fragmentation, increased network length, stabilized mitochondrial membrane potential, enhanced ATP production, and lowered ROS accumulation under oxidative stress. Metformin significantly increased mitophagy and autophagic flux, as shown by LC3B puncta quantification with and without chloroquine, and activated AMPK signaling through increased AMPKα1/2 phosphorylation and AMPKβ1 Ser182 phosphorylation. In addition, metformin promoted PGC‐1α nuclear translocation, indicating stimulation of mitochondrial biogenesis, while maintaining mtDNA copy number and supporting oxidative phosphorylation. These findings suggest that metformin, at clinically relevant concentrations, enhances mitochondrial health and function in LHON fibroblasts, supporting its potential as an affordable and safe therapeutic option to mitigate vision loss in LHON.

## Graphical abstract

Metformin mediates mitochondrial quality control in Leber's hereditary optic neuropathy (LHON) fibroblasts carrying mtDNA mutations. At therapeutic levels, metformin activates AMPK signaling to restore mitochondrial dynamics by promoting fusion and restraining fission, while preserving mitochondrial mass, enhancing autophagy/mitophagy and biogenesis, reducing ROS, and improving ATP production and membrane potential. Together, these actions support overall mitochondrial health.

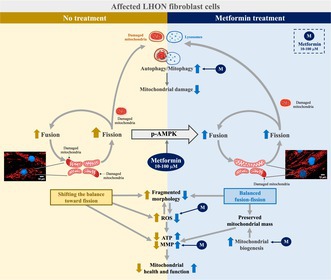

**Abbreviations**
AMPK: AMP‐activated protein kinase
ATP: Adenosine triphosphate
CQ: Chloroquine
DRP1: Dynamin‐related protein 1
FCCP: Carbonyl cyanide‐p‐trifluoromethoxyphenylhydrazone
H_2_O_2_
: Hydrogen peroxide
LC3B: Microtubule‐associated protein 1 light chain 3B
LHON: Leber's hereditary optic neuropathy
MMP: Mitochondrial membrane potential
mtDNA: Mitochondrial DNA
mTOR: Mammalian target of rapamycin
mTORC1: Mammalian target of rapamycin complex 1
OCR: Oxygen consumption rate
OCTs: Organic cation transporters
p70(S6K): Phospho‐p70 S6 kinase
PBMCs: Peripheral blood mononuclear cells
PEN2: Presenilin enhancer 2
PGC1α: Peroxisome proliferator‐activated receptor‐gamma coactivator 1‐alpha
RGCs: Retinal ganglion cells
ROS: Reactive oxygen species
RPE: Retinal pigment epithelial
ULK1: Unc‐51 like autophagy activating kinase 1

Leber's hereditary optic neuropathy (LHON) is a maternally inherited disease and the most prevalent mitochondrial disorder in Thailand. It primarily results from mitochondrial DNA (mtDNA) mutations, with approximately 95% of patients linked to mutations at m.11778G>A, m.3460G>A, and m.14484 T>C. These mutations affect the MT‐ND4, MT‐ND1, and MT‐ND6 protein subunits of complex I in the mitochondrial respiratory chain, leading to decreased adenosine 5′‐triphosphate (ATP) production and increased reactive oxygen species (ROS) [1]. This chain of events profoundly affects retinal ganglion cells (RGCs), which are highly dependent on ATP due to their high mitochondrial content, ultimately causing degeneration of RGCs and central vision loss [2]. While idebenone offers some therapeutic benefit [3], its limited accessibility in many countries underscores the pressing need to develop accessible alternative therapies.

Mitochondrial dynamics, the continuous cycles of fusion and fission, are essential for maintaining mitochondrial health. Fusion promotes the formation of interconnected tubular networks that allow the exchange of mitochondrial proteins and mtDNA, helping to dilute damaged components and support efficient oxidative phosphorylation [4]. Fission, in contrast, segregates defective mitochondria for degradation through mitophagy (mitochondrial autophagy), thereby sustaining cellular function [5]. Excessive fission, however, can disrupt this balance, driving ROS accumulation, mitochondrial membrane potential (MMP) collapse, and ATP depletion [6]. These changes compromise mitochondrial function and contribute to oxidative stress, a hallmark of mitochondrial disorders such as LHON, that has been linked to these imbalances [7]. Achieving a proper equilibrium in mitochondrial dynamics is essential to preserve mitochondrial quality and prevent dysfunction [8].

Recent studies have focused on improving mitochondrial dynamics as a promising therapeutic strategy. Metformin, a widely used and affordable medication, has emerged as a potential candidate for repurposing. It has an oral bioavailability of 40–60%, is completely absorbed within 6 h, distributes rapidly into tissues, and is excreted unchanged by the kidneys [9]. Following standard oral doses of 1000–2000 mg, plasma concentrations typically range from 2.8 to 40 μm, with higher levels in tissues than in plasma [10]. Metformin crosses the blood–brain barrier, reaching concentrations in cerebrospinal fluid levels that are two‐ to threefold higher than those in plasma [11]. It enters cells mainly via organic cation transporters (OCTs). Mechanistically, metformin activates AMP‐activated protein kinase (AMPK), suppressing hepatic glucose production and modulating insulin signaling pathways [12]. Beyond its metabolic effects, metformin has been shown to improve mitochondrial health in diverse human cell types. It regulates mitochondrial dynamics by upregulating fusion proteins and downregulating fission proteins, enhancing mitochondrial function in leukocytes from diabetic patients treated with metformin for 1 year [13]. In addition, metformin promotes mitochondrial health and activity in peripheral blood mononuclear cells through AMPK‐dependent mitophagy [14] and improves mitochondrial function in fibroblasts obtained from fetuses with Down syndrome [15]. It also activates the lysosomal AMPK signaling via presenilin enhancer 2 (PEN2), promoting mitochondrial biogenesis, mitophagy, and energy homeostasis [16], and reduces ROS production through an AMPK‐dependent antioxidant pathway [17, 18]. Together, these findings open a new avenue for exploring the role of metformin in treating mitochondrial disorders such as LHON.

Despite its established metabolic benefits, the effects of metformin on mitochondrial function in LHON remain largely unexplored. This study addresses this gap by investigating the therapeutic potential of metformin in primary skin fibroblasts derived from patients carrying the m.11778G>A mutation. Fibroblasts provide a practical and informative model, as their mitochondrial content and dynamics resemble those of neuronal axons [19, 20]. Using high‐content imaging and quantitative analysis with CellProfiler [21], we assess the effects of metformin on mitochondrial morphology, dynamics, autophagy/mitophagy, and overall health in fibroblasts from both LHON patients and healthy controls. By focusing on these parameters, this study seeks to generate new insights into how metformin may improve mitochondrial quality control, offering a potential strategy to mitigate vision loss in LHON patients.

## Materials and methods

### Sample collection and selection

Patients with confirmed blindness due to LHON were recruited through the Ophthalmology Department at Siriraj Hospital. Primary skin fibroblasts were established from these patients and compared with fibroblasts from three unrelated healthy individuals without a family history of blindness. All procedures followed the ethical principles of the Declaration of Helsinki (1964) and were approved by the Human Research Protection Unit of Mahidol University (Certificate of Approval No. Si 161/2019). All participants provided written informed consent for the use of their samples in research. For patients with visual impairment due to LHON, the full consent form was read aloud to them before they provided their written signature. Details of patients and control fibroblast samples are provided in Table S1.

### Culture system for primary human fibroblast cells

Primary human fibroblasts (Passages 1–6) were cultured in Dulbecco's Modified Eagle Medium containing 5 mm glucose (Sigma‐Aldrich, St. Louis, MO, USA) supplemented with 10% fetal bovine serum (FBS, Sigma‐Aldrich), 100 U·mL^−1^ penicillin, 100 μg·mL^−1^ streptomycin (Sigma‐Aldrich), and 2.5 μg·mL^−1^ amphotericin B (Bio Basic Inc., Markham, ON, Canada). Cells were maintained at 37 °C in a humidified incubator with 5% CO_2_.

### Drug treatment

Metformin hydrochloride (Glentham Life Sciences, Corsham, Wiltshire, UK) was prepared in culture medium to final concentrations of 5, 10, 50, 100, 500, and 1000 μm. Fibroblasts were treated for 24 or 48 h. For stress assays, cells were pretreated with metformin for 24 h, followed by exposure to 50 μm phenanthroline (Sigma‐Aldrich) for 4 h to assess mitochondrial fragmentation or 200 μm H_2_O_2_ for 1 h to induce ROS production.

### Mitochondrial fragmentation and length measurement

Fibroblasts were seeded at a density of 5000 cells per well in a 96‐well plate (PerkinElmer, Waltham, MA, USA) and stained for 30 min at 37 °C with MITO‐ID^®^ Red (1 : 10000; Enzo Life Sciences, Farmingdale, NY, USA) to label mitochondria and Hoechst 33342 (1 : 1000; Tocris Bioscience, Bristol, UK) to stain nuclei. Images were acquired using the Operetta CLS™ high‐content imaging system with the following filter settings: λ_ex_ 530–560 nm/λ_em_ 655–705 nm for MITO‐ID^®^ Red, and λ_ex_ 355–385 nm/λ_em_ 430–500 nm for Hoechst. Mitochondrial fragmentation and length were quantified using CellProfiler software (Broad Institute, Cambridge, MA, USA) following established methods [22].

### ATP measurement

Total cellular ATP levels were assessed using the ATPlite 1 step luminescent assay system (PerkinElmer). Fibroblasts were seeded at a density of 10 000 cells per well in 96‐well plates (Corning^®^, Corning, NY, USA). After drug treatments, the substrate solution was added directly to the wells, and luminescence was measured using a microplate reader (BioTek, Winooski, VT, USA). Results were expressed as a percentage of ATP content relative to untreated control cells.

### Mitochondrial membrane potential (MMP) measurement

Fibroblasts were seeded at a density of 5000 cells per well and stained for 45 min at 37 °C with JC‐10 (1 : 100, ultra‐pure; Enzo Life Sciences) and Hoechst 33342 (1 : 1000). JC‐10 orange‐fluorescent aggregates were detected using the Rhodamine filter set (λ_ex_ 530–560 nm/λ_em_ 570–650 nm), while JC‐10 green‐fluorescent monomers were detected using the Alexa 488 filter set (λ_ex_ 460–490 nm/λ_em_ 500–550 nm). MMP status was determined by calculating the ratio of orange to green fluorescent intensity, normalized to nuclear staining [22].

### Mitochondrial H_2_O_2_ measurement

Fibroblasts were seeded at 5000 cells per well and stained for 45 min at 37 °C with MitoPY1 (1 : 1000; Tocris Bioscience) to detect hydrogen peroxide (H_2_O_2_), MITO‐ID^®^ Red (1 : 10000), and Hoechst 33342 (1 : 1000). MitoPY1 fluorescence was detected at λ_ex_ 490–515 nm/λ_em_ 525–580 nm. A customized CellProfiler pipeline was used to enhance sensitivity by correlating green‐speckled MitoPY1 fluorescence with mitochondrial and nuclear markers [22].

### Mitophagy measurement

Fibroblasts at a density of 5000 cells per well were stained for 30 min at 37 °C with LysoGreen (1 : 625; Abcam, Cambridge, UK) to label lysosomes, MITO‐ID^®^ Red (1 : 1000), and Hoechst 33342 (1 : 1000). LysoGreen fluorescence was measured at λ_ex_ 435–460 nm/λ_em_ 470–515 nm alongside MITO‐ID^®^ Red and Hoechst. Mitophagy levels were assessed using the skeletonized technique in CellProfiler version 4.0.7 by identifying colocalization between mitochondrial and lysosomal skeletons. The proportion of colocalized area, normalized by total mitochondria, was used as a measure of mitophagy [23]. Representative processed images from each analysis step are shown in Fig. S1, and detailed methods will be described separately.

### Western blot analysis

Fibroblasts were cultured in T25 flask (500 000 cells) and harvested for protein extraction using Mammalian‐PE™ kit (Gold Biotechnology, St. Louis, MO, USA). Proteins were separated by SDS/polyacrylamide gel electrophoresis (SDS/PAGE) and transferred to polyvinylidene difluoride (PVDF) membranes (PALL Life Sciences, Port Washington, NY, USA). Membranes were probed with primary antibodies: anti‐phosphorylated (phospho)‐AMPKα1/2 at Thr183/Thr172 (ab133448, 1 : 1000, Abcam), anti‐AMPKα1/2 (ab207442, 1 : 2000, Abcam), anti‐LC3B (2775, 1 : 1000, Cell Signaling, Danvers, MA, USA), anti‐phospho‐DRP1 at Ser616 (3455, 1 : 1000, Cell Signaling), anti‐DRP1 (5391, 1 : 1000, Cell Signaling), and anti‐β‐actin (4970, 1 : 2000, Cell Signaling). After incubation with horseradish peroxidase (HRP)‐conjugated secondary antibodies (ab6721, 1 : 20000, Abcam), protein bands were visualized using Enhanced Chemiluminescence (BioRad Laboratories, Hercules, CA, USA) and quantified by the ImageJ software (NIH, Bethesda, MD, USA).

### Mitochondrial mass measurement

Fibroblasts (500 000 cells per T25 flask) were harvested and centrifuged for DNA extraction using the Genomic DNA Prep Kit (BioFact, Daejeon, Republic of Korea). Mitochondrial mass was assessed by quantifying mtDNA copy number through the mitochondrially encoded tRNA leucine 1 (*MT‐TL1*) gene, normalized to nuclear DNA copy number using the *ZHX2* gene. Quantification was performed by real‐time PCR with the SYBR Green PCR master mix (Roche, Basel, Switzerland) on the LightCycler^®^480 (Roche). Primer sequences for *MT‐TL1* and *ZHX2* genes are detailed in Table S2. Relative mtDNA content was determined using the ΔΔ*C*_t_ method.

### PGC‐1α, p70(S6K), pAMPK, and LC3B—immunofluorescence and quantification

Fibroblasts (5000 cells per well) were fixed in 4% formaldehyde for 15 min at room temperature, permeabilized with cold absolute methanol for 5 min at −20 °C, and blocked for 1 h (Cell Signaling immunofluorescence blocking buffer; 3% BSA for AMPK staining). Cells were incubated overnight at 4 °C with primary antibodies: CoraLite^®^ Plus 488‐conjugated anti‐PGC‐1α (Proteintech, Rosemont, IL, USA), anti‐phospho‐p70(S6K) (Thr389; Proteintech), anti‐pAMPK β1 (Ser182; Proteintech), or anti‐LC3B (Cell Signaling). For p70(S6K), pAMPK, and LC3B, staining was completed with a CoraLite^®^ Plus 488‐conjugated secondary antibody (Proteintech). Nuclei were counterstained with DAPI (10 min, room temperature). Images were acquired on the Operetta CLS™ using filter sets λ_ex_ 460–490 nm/λ_em_ 500–550 nm (CoraLite 488) and λ_ex_ 355–385 nm/λ_em_ 430–500 nm (DAPI). Quantification was performed in CellProfiler: Nuclei were segmented from DAPI; cell boundaries were generated; and cytoplasmic masks were derived by subtracting the nuclei. Mean cytoplasmic intensities were measured for p70(S6K), pAMPKβ1, and LC3B. LC3B puncta were counted by enhancing edges, then segmenting puncta with a conservative lower‐bound threshold to minimize false positives. For PGC‐1α, nuclear CoraLite‐488 signal was classified per nucleus (high‐intensity = nuclear translocation; low‐intensity = no translocation) to report the percentage of PGC‐1α‐positive nuclei. Detailed methods for image analysis with CellProfiler are provided in the Supporting Information.

### Statistical analysis

Statistical comparisons were performed using one‐way or two‐way analysis of variance (ANOVA), followed by Tukey's post hoc test where appropriate. Differences between two groups were assessed using the Student's independent sample *t*‐test. All analyses were conducted in the Jamovi software (Jamovi project, Sydney, NSW, Australia) and in R program (R Core Team, Vienna, Austria). Data were presented as means with 95% confidence intervals (CI). Statistical significance was set at *P < 0.05, **P < 0.01, and ***P < 0.001. Graphs were generated using the Prism software (GraphPad Software Inc., San Diego, CA, USA).

## Results

### Mitochondria in LHON fibroblasts fragmented more, had lower MMP, and produced less ATP

Previous studies have demonstrated that LHON mutations impair complex I activity, resulting in diminished ATP synthesis, decreased MMP, and altered ROS production in patient‐derived cells [24]. In this study, fibroblasts from LHON patients were screened for three primary pathogenic variants. Sequencing confirmed a homoplasmic m.11778G>A substitution on the *MT‐ND4* gene (Fig. S2), while the *MT‐ND1* (m.3460G>A) and *MT‐ND6* (m.14484 T>C) retained the reference bases (G and T, respectively) (Figs S3 and S5). The m.11778G>A mutation was present in all mtDNA copies of the LHON fibroblasts [25]. Conversely, fibroblasts from healthy controls exhibited reference at all three sites (Figs S2, S4, and S6).

**Fig. 1:**
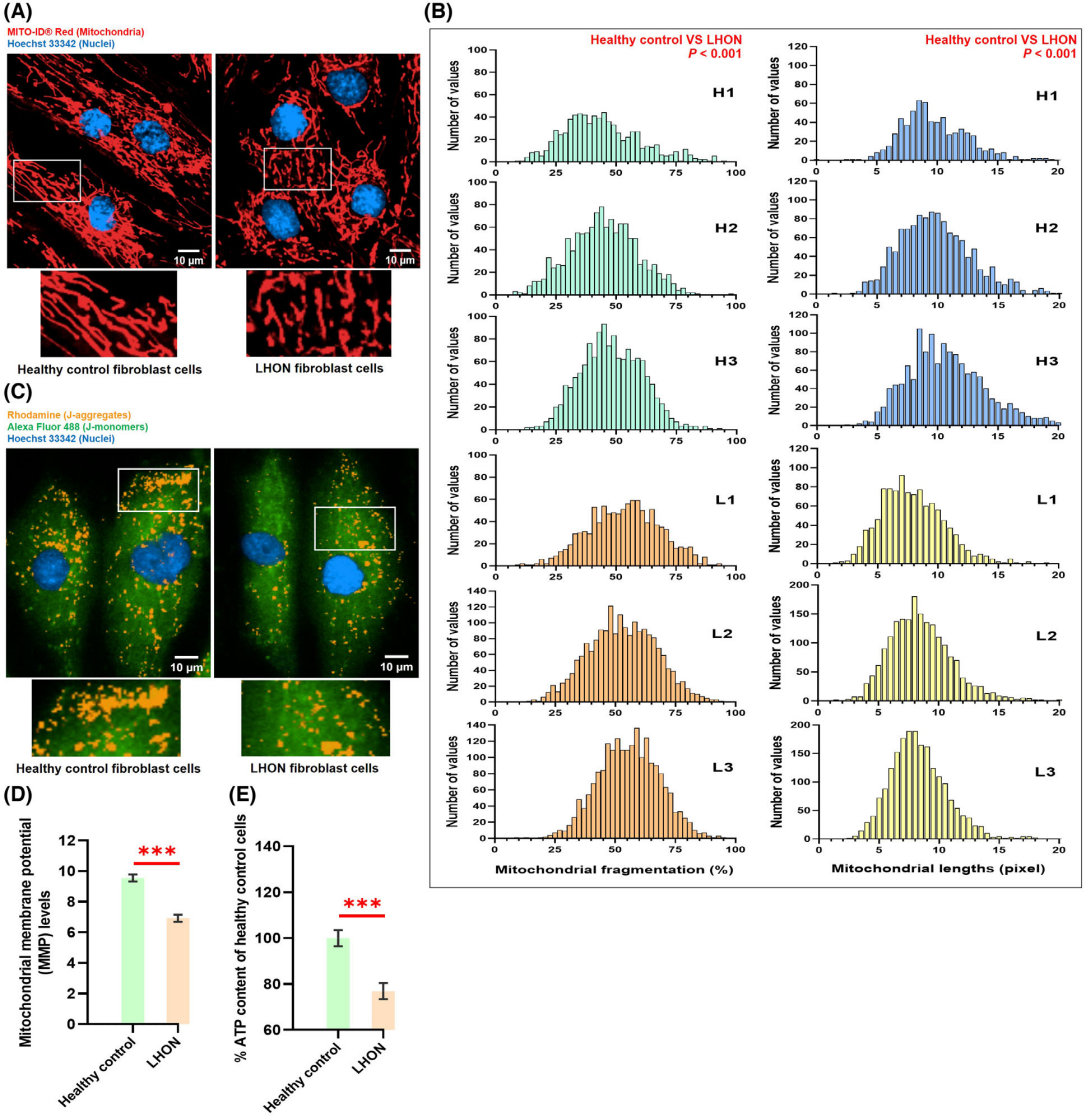
LHON fibroblasts exhibit mitochondrial fragmentation, reduced membrane potential, and lower ATP levels. (A) MITO‐ID® Red staining reveals increased mitochondrial fragmentation in LHON fibroblasts compared with healthy controls. (B) Quantitative analysis shows higher fragmentation (%) and shorter mitochondrial length (pixels per cell) in LHON fibroblast samples (L1‐L3) compared with healthy fibroblasts (H1‐H3). (C) JC‐10 fluorescent images show reduced orange (rhodamine) aggregates in LHON fibroblasts, indicating decreased polarized mitochondrial membrane potential (MMP). (D) Quantitative analysis confirms a significant decrease in MMP per cell in LHON fibroblasts relative to healthy controls. (E) Luminescence‐based ATP assay shows lower cellular ATP content (%) in LHON fibroblasts, normalized to 100% in controls. Images were captured using an Operetta CLS™ system with a 40× objective (scale bar: 10 μm). Each sample was imaged across 64–72 fields per well (15–40 cells per field), yielding 1900–5700 cells per sample (duplicate wells). The bar graphs represent mean ± 95% CI. Statistical comparisons were performed using the Student's *t*‐test. Significance: *P < 0.05, **P < 0.01, and ***P < 0.001.

Compared with healthy controls, LHON fibroblasts displayed a more fragmented mitochondrial network with shorter branches and fewer interconnections (Fig. 1A). Distribution analyses of individual patient samples confirmed a higher proportion of fragmented mitochondria and shorter average mitochondrial lengths (in pixels) per cell (Fig. 1B). On average, mitochondrial fragmentation increased by 8.3% with mean mitochondrial length reduced to 1.93 pixels (~0.19 μm), relative to healthy controls (Fig. S7).

MMP polarization was also reduced in LHON fibroblasts with values dropping to 2.62 compared to healthy controls (Fig. 1C,D). In line with these changes, ATP production was significantly impaired, showing a 23.1% reduction relative to healthy controls (Fig. 1E). These results clearly indicate that the m.11778G>A mutation compromises mitochondrial integrity and bioenergetics in LHON fibroblasts, producing a shorter and more fragmented mitochondrial network, lower MMP, and decreased ATP content. These findings highlight the central role of this mutation in mitochondrial dysfunction and underscore the need for therapeutic interventions aimed at restoring mitochondrial function in LHON patients.

### Metformin reduced mitochondrial fragmentation, promoted mitochondrial length, and mitigated against stress‐induced fragmentation in healthy control and LHON fibroblasts

**Fig. 2:**
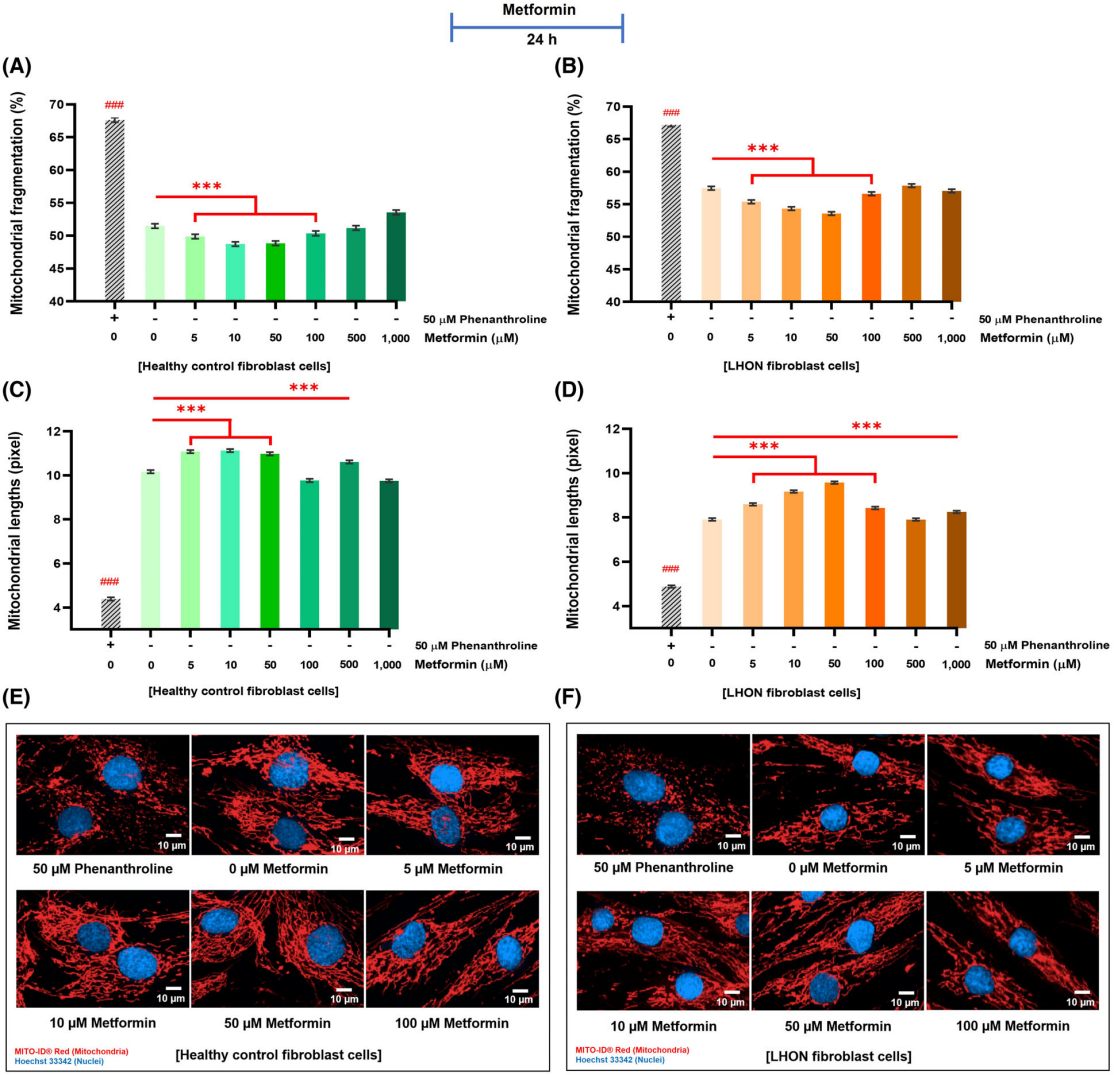
Metformin attenuates mitochondrial fragmentation and promotes network elongation in healthy and LHON fibroblasts. (A, B) Treatment with metformin (5–100 μm, 24 h) significantly reduced mitochondrial fragmentation (%) in both healthy and LHON fibroblasts compared with untreated controls. (C, D) Mitochondrial length (pixels per cell) increased following metformin exposure, notably at 5–50 μm in healthy fibroblasts and 5–100 μm in LHON fibroblasts. (E, F) Representative MITO‐ID® Red images show more elongated and interconnected mitochondrial networks after metformin treatment. Phenanthroline (50 μm) was used as a positive control for fragmentation. Images were obtained using Operetta CLS™ (40× objective; scale bar = 10 μm) across 60–80 fields per well (1800–6400 cells per condition). Data are expressed as mean ± 95% CI (*n* = 3 per group; ≥ 3 independent experiments). Two‐way ANOVA with Tukey's post‐test: *P < 0.05, **P < 0.01, and ***P < 0.001 versus untreated; ### denotes phenanthroline versus control.

Mitochondrial morphology is closely linked to mitochondrial function and overall cellular health [26]. To evaluate the effects of metformin, phenanthroline was used as a positive control to induce mitochondrial fragmentation [27]. Metformin treatment at concentrations of 5–100 μm significantly reduced mitochondrial fragmentation in both healthy control fibroblasts (Fig. 2A,E) and LHON fibroblasts (Fig. 2B,F). Notably, the most pronounced increase in mitochondrial length was observed in healthy fibroblasts treated with 10 μm metformin, showing an average increase of 0.96 pixels (Fig. 2C,E). In LHON fibroblasts, mitochondrial length increased by up to 1.67 pixels following treatment with 50 μm metformin (Fig. 2D,F). Individual patient data are presented in Fig. S8.

**Fig. 3:**
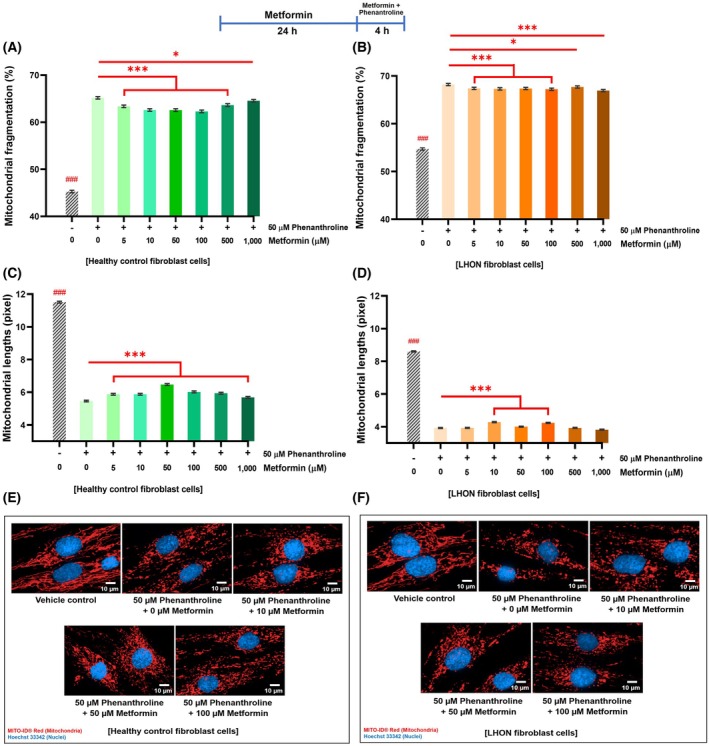
Metformin prevents phenanthroline‐induced mitochondrial fragmentation in healthy control and LHON fibroblasts. (A, B) Metformin (24 h) significantly reduced mitochondrial fragmentation induced by 50 μm phenanthroline (4 h). (C, D) Mitochondrial length (pixels per cell) increased in fibroblasts treated with metformin during phenanthroline exposure. (E, F) MITO‐ID® Red fluorescence shows that metformin preserves network structure, with fewer small fragments under stress conditions. Vehicle control: 0.2% ethanol. Imaging: Operetta CLS™ (40× objective; scale bar = 10 μm). Sampling: 60–80 fields per well, 15–40 cells per field (1800–6400 cells per condition, duplicates). Data: mean ± 95% CI (*n* = 3 per group; ≥ 3 independent experiments). Two‐way ANOVA with Tukey's post‐test: *P < 0.05, **P < 0.01, and ***P < 0.001 versus phenanthroline; ### denotes control versus phenanthroline.

The effect of metformin against stress‐induced mitochondrial fission was next examined. Cotreatment with metformin and phenanthroline significantly reduced mitochondrial fragmentation compared with phenanthroline alone, in both healthy control (Fig. 3A,E) and LHON fibroblasts (Fig. 3B,F). In healthy controls, metformin at 5–1000 μm significantly diminished phenanthroline‐induced shortening of the mitochondrial network (Fig. 3C,E). In LHON fibroblasts, protective effects were most evident at 10–100 μm (Fig. 3D,F). Fig. S9 provides individual sample data demonstrating metformin's protective action.

Metformin also reduced activation of DRP1, a key protein of mitochondrial fission, in the L2 fibroblast sample. Treatment with 50 μm metformin for 24 h significantly decreased the p‐DRP1/total DRP1 ratio, whereas no significant changes were observed in the other LHON samples (Fig. S10).

Overall, metformin consistently reduced mitochondrial fragmentation and promoted a longer mitochondrial network under both basal conditions and phenanthroline‐induced stress. Effective concentrations for maintaining mitochondrial health ranged between 10 and 100 μm, though a biphasic effect was noted, with adverse morphological changes observed at concentrations above 100 μm.

### Metformin improved cellular energy status and mitochondrial membrane potential in healthy control and LHON fibroblasts

LHON mutations impair complex I‐dependent ATP production [28] and disrupted mitochondrial dynamics can further exacerbate this deficit [6]. Since mitochondria are the primary site of ATP production, measuring cellular ATP levels provides a useful readout of mitochondrial function. In this study, we assessed the effects of metformin on ATP content in healthy control and LHON fibroblasts using a luminescence‐based assay.

**Fig. 4:**
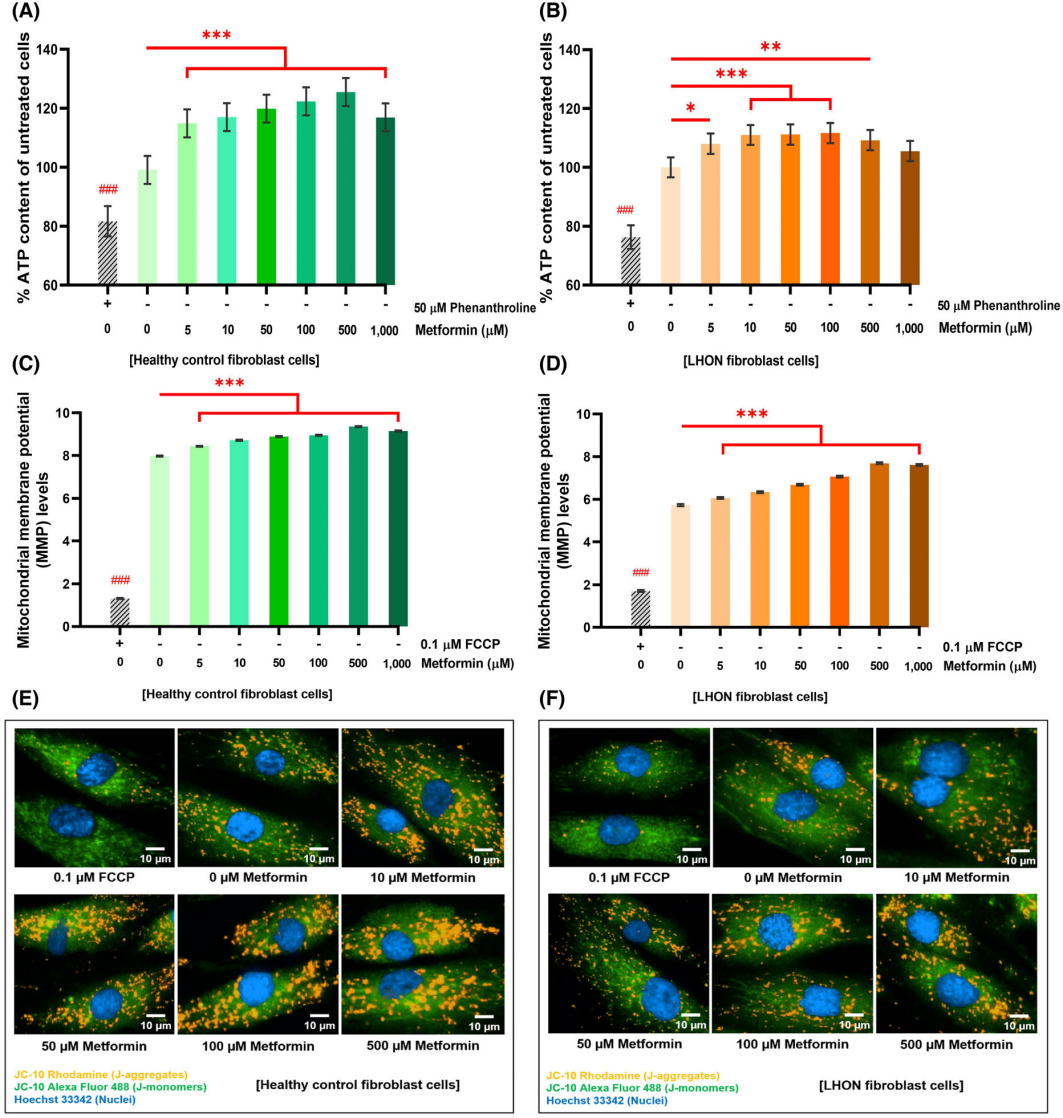
Metformin enhances ATP production and mitochondrial membrane potential in healthy control and LHON fibroblasts. (A, B) ATPlite® luminescence assay shows increased cellular ATP (%) after 24‐h metformin treatment in both healthy and LHON fibroblasts. Phenanthroline (50 μm) decreased ATP content relative to controls. (C, D) MMP levels, measured as the JC‐10 orange/green fluorescence ratio, significantly increased with 5–1000 μm metformin. (E, F) Representative JC‐10 fluorescence images show higher orange aggregates, indicating enhanced mitochondrial polarization after metformin treatment. FCCP (0.1 μm) served as a positive depolarization control. Imaging: 40× objective; scale bar = 10 μm. Sampling: 65–75 fields per well, 15–35 cells per field (1950–5250 cells per condition, duplicates). *n* = 3 per group; ≥ 3 independent trials per sample (controls = 11, LHON = 13 for ATP; controls = 9, LHON = 9 for MMP). Data: mean ± 95% CI. Two‐way ANOVA with Tukey's post‐test: *P < 0.05, **P < 0.01, and ***P < 0.001 versus untreated; ### denotes phenanthroline or FCCP versus untreated.

Metformin significantly enhances ATP synthesis in both groups. In healthy fibroblasts, the maximal effect was observed at 500 μm, where ATP levels rose by 26.4% compared to untreated controls (Fig. 4A). In LHON fibroblasts, ATP content increased across 5–500 μm, with the most pronounced improvement of 11.7% at 100 μm (Fig. 4B). At higher concentrations (500 μm and 1000 μm), a biphasic pattern emerged, with ATP levels slightly decreased, consistent with the adverse morphological effects at suprapharmacological doses. Fig. S11 shows the effect of metformin on ATP levels in individual LHON samples.

MMP, another critical marker of mitochondrial function, reflects the electrochemical gradient across the inner mitochondrial membrane that drives ATP synthesis. Loss of MMP is a hallmark of mitochondrial dysfunction [29] and is commonly reported in LHON. Using JC‐10 staining, polarized mitochondria were detected as orange J‐aggregates, whereas depolarized mitochondria appeared as green monomers [30, 31]. Metformin treatment significantly increased MMP in both healthy control (Fig. 4C,E) and LHON fibroblast cells (Fig. 4D,F) across a concentration range from 5 to 1000 μm for 24 h. Similar to ATP results, MMP levels decreased slightly at 1000 μm compared to 500 μm. Fig. S12 displays results for the individual LHON samples.

To further assess mitochondrial function, we measured oxygen consumption rates in LHON fibroblasts using the Seahorse XFp Analyzer at 50 and 100 μm metformin. Parameters, including basal ATP‐linked, and maximal respiration, as well as spare capacity, and coupling efficiency, were not impaired (Figs S13–S15). In the L3 fibroblasts, 50 μm metformin even increased basal and ATP‐linked respiration (Fig. S16).

Together, these findings indicate that metformin enhances ATP production and stabilizes mitochondrial membrane potential without compromising oxidative phosphorylation.

### Metformin reduced mitochondrial ROS levels in healthy control and LHON fibroblasts under stress conditions

In LHON, retinal ganglion cell mitochondria exhibit elevated ROS production alongside impaired antioxidant defenses. ROS accumulation contributes directly to disease pathogenesis [32] by promoting excessive mitochondrial fission and cellular damage [6]. Maintaining a balance between ROS generation and elimination is therefore crucial for mitochondrial health. Metformin is known to activate the AMPK‐FOXO3 signaling pathway, reducing ROS generation in many cell types [18].

**Fig. 5:**
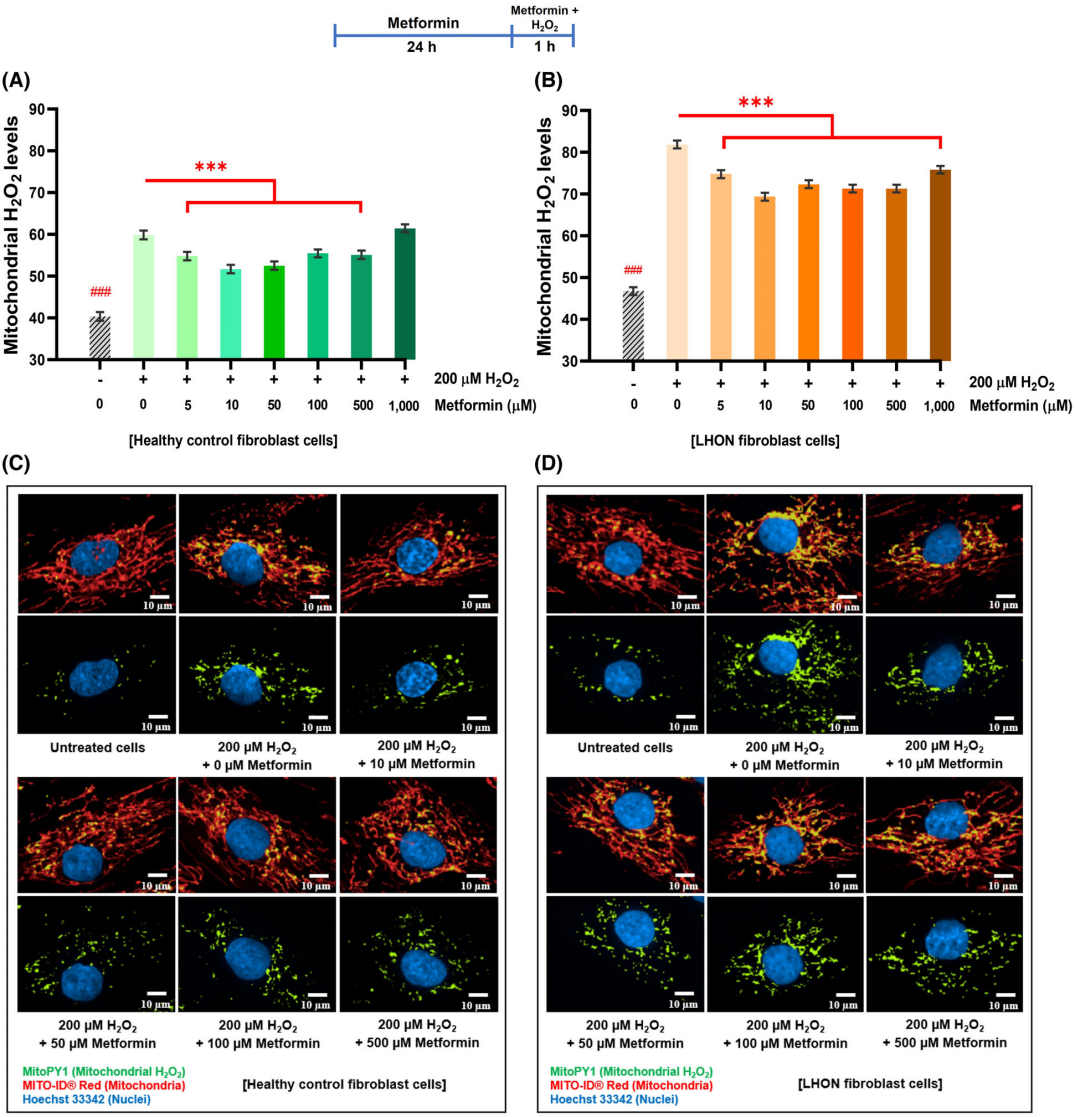
Metformin mitigates H_2_O_2_‐induced mitochondrial oxidative stress in healthy controls and LHON fibroblasts. Fibroblasts were pretreated with metformin (24 h) and then exposed to 200 μm H_2_O_2_ (1 h) in the presence of metformin. (A, B) Quantification of mitochondrial H_2_O_2_ levels was performed by analyzing colocalization of MitoPY1 (green) and MITO‐ID® Red fluorescence relative to nuclei. Metformin markedly reduced mitochondrial H_2_O_2_ accumulation under oxidative stress. (C, D) Merged fluorescence images confirm reduced green‐speckled signal after metformin treatment. Imaging: 40× objective; scale bar = 10 μm. Sampling: duplicate wells, 50–60 fields per well (15–35 cells per field; 1500–4200 cells per condition). *n* = 3 per group; ≥ 3 independent experiments. Data: mean ± 95% CI. Two‐way ANOVA with Tukey's post‐test: *P < 0.05, **P < 0.01, and ***P < 0.001 versus H_2_O_2_ alone; ### denotes untreated versus H_2_O_2_.

To test whether metformin reduces mitochondrial ROSs, we used the MitoPY1 fluorescent probe, which selectively detects mitochondrial hydrogen peroxide H_2_O_2_ [33]. Under oxidative stress induced by 200 μm H_2_O_2_, metformin treatment significantly lowered mitochondrial H_2_O_2_ levels in both healthy fibroblasts (5–500 μm, Fig. 5A), and LHON fibroblasts (5–1000 μm, Fig. 5B), with the strongest reduction at 10 μm. No consistent dose–response relationship was observed, a pattern similar to that seen for metformin's effect on mitochondrial morphology and ATP production.

Fluorescence imaging further confirmed that metformin attenuated ROS accumulation. In both healthy and LHON fibroblasts, treatment with 10, 50, 100, and 500 μm metformin reduced colocalization between green MitoPY1 signals (H_2_O_2_) and red mitochondrial staining compared with cells exposed to H₂O₂ alone (Fig. 5C,D). Control cells without H_2_O_2_ or metformin showed minimal green‐speckled fluorescence, validating the ROS‐inducing effect of H_2_O_2_. Results for individual LHON samples are shown in Fig. S17. These findings suggest that metformin reduces mitochondrial ROS levels under oxidative stress, supporting its potential to alleviate oxidative stress in LHON fibroblasts.

### Metformin enhanced mitophagy and autophagy in LHON fibroblasts

Mitophagy is a selective removal of damaged mitochondria through their encapsulation by phagophores and subsequent degradation in lysosomes [34]. To examine the effect of metformin on mitophagy, fibroblasts were stained with Hoechst 33342 for nuclei, MITO‐ID^®^ Red for mitochondria, and LysoGreen for lysosomes. The images were analyzed to determine the extent of mitochondria‐lysosome colocalization, which was normalized with the mitochondria count in each cell [23].

**Fig. 6:**
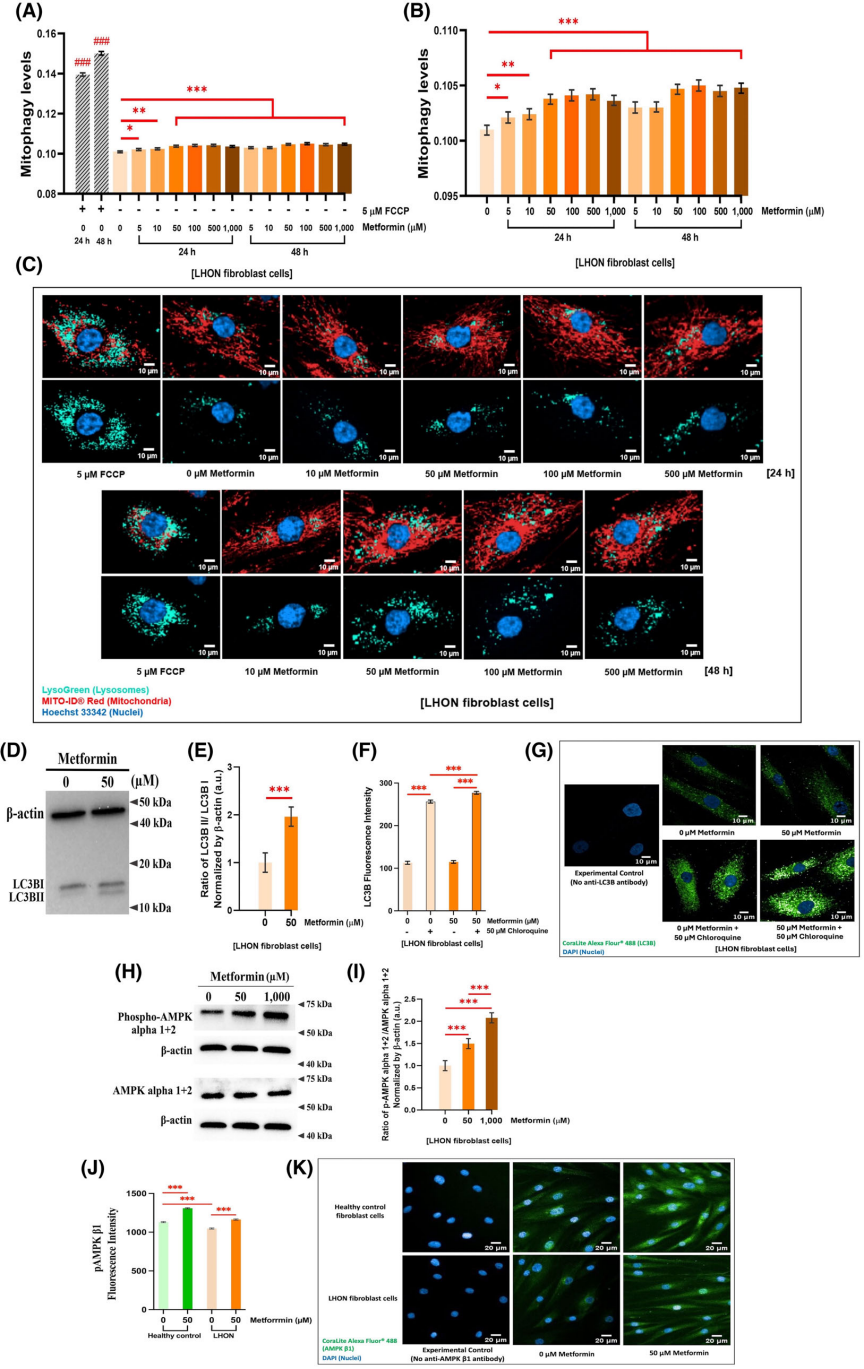
Metformin enhances mitophagy and activates AMPK signaling in LHON fibroblasts. (A, B) Quantitative analysis shows that metformin treatment for 24 and 48 h significantly increased mitophagy levels in LHON fibroblasts compared with untreated cells. FCCP served as a positive control. (C) Representative fluorescence images demonstrate increased colocalization between mitochondria and lysosomes following metformin treatment, indicating enhanced mitophagy. (D, E) Immunoblot analysis shows that 50 μm metformin (24 h) elevated LC3B‐II levels, an autophagic marker, in LHON fibroblasts. β‐Actin served as a loading control. (F) Quantification of LC3B puncta fluorescence intensity in LHON fibroblasts treated with 50 μm metformin with or without 50 μm chloroquine (CQ) for 24 h. Metformin increased LC3B puncta formation, and further accumulation in the presence of CQ indicates increased autophagic flux. (G) Representative immunofluorescence images of LC3B (green) and nuclei (DAPI, blue) show enhanced puncta formation in metformin‐treated LHON fibroblasts. The experimental control without primary antibody confirmed signal specificity. (H) Representative immunoblots of phospho‐AMPKα1/2 (Thr183/Thr172) and total AMPKα1/2 in LHON fibroblasts treated with 50 and 1000 μm metformin for 24 h. β‐Actin was used as a loading control. (I) Quantification of phospho‐AMPKα1/2 to total AMPKα1/2 ratios normalized to β‐Actin. (J) Quantification of AMPKβ1 phosphorylation (Ser182) fluorescence intensity normalized to DAPI in healthy control and LHON fibroblasts treated with 50 μm metformin for 24 h, showing a significant increase in both groups. (K) Representative immunofluorescence images of AMPKβ1 (green) and nuclei (DAPI, blue) in healthy control and LHON fibroblasts following metformin treatment. Mitophagy assay: Imaging was performed using the Operetta CLS™ high‐content system (20× objective; scale bar, 10 μm). Sampling: 35–50 Fields per well, 160–180 cells per field (11 200–18 000 cells per condition, in duplicate). Independent replicates: 10 for mitophagy, 9 for LC3B immunoblotting, and 10 for AMPK immunoblotting. LC3B puncta and AMPKβ1 imaging: 40× Objective; scale bar, 20 μm. Sampling: 25–45 Fields per well, 2500–9000 cells per condition, in duplicate. Data are presented as mean ± 95% CI (*n* = 3 per group; ≥ 3 independent experiments). Statistical analysis was performed using two‐way ANOVA with Tukey's post‐test or Student's *t*‐test. *P < 0.05, **P < 0.01, ***P < 0.001 versus untreated cells; ### indicates FCCP versus untreated controls. a.u., arbitrary units.

Metformin treatment significantly increased mitophagy in LHON fibroblasts compared with untreated controls, as indicated by greater mitochondria‐lysosome colocalization normalized to mitochondria count (Fig. 6A,B). The effects varied with concentration and treatment duration, with the largest increases observed at 1000 μm for 24 h and 500 μm for 48 h. Responses differed across individual patient samples as shown in Fig. S18. Fluorescent imaging further confirmed enhanced mitochondrial and lysosomal colocalization, indicating in LHON fibroblasts treated with 10, 50, 100, and 500 μm metformin for 24 and 48 h (Fig. 6C).

Metformin also activated autophagy in LHON fibroblasts. This was demonstrated by increased conversion of LC3B‐I to LC3B‐II, a marker of autophagosome formation [35], following treatment with 50 μm metformin for 24 h (Fig. 6D,E). Detailed responses in individual LHON fibroblasts samples are provided in Fig. S19. To further assess autophagic flux, LC3B puncta were quantified by immunofluorescence in fibroblasts treated with metformin alone or in combination with chloroquine (CQ). Metformin reduced the proportion of cells lacking puncta and significantly increased LC3B puncta fluorescence intensity, while additional accumulation in the presence of CQ confirmed ongoing autophagosome formation and flux rather than a block in degradation (Fig. 6F,G). Results and calculations for autophagic flux are shown in Table S3.

These results suggest that by promoting both mitophagy and autophagy, metformin supports mitochondrial quality control, facilitating the removal of damaged mitochondria associated with the LHON mutation.

AMP‐dependent protein kinase (AMPK), a central energy sensor, regulates stress response and mitochondrial quality control. Its activation through phosphorylation at threonine residues in the α subunits promotes mitochondrial quality control. Metformin is known to target AMPK [12], which in turn protects against stress‐induced mitochondrial dysfunction [36], regulates mitochondrial dynamics [37], and promotes mitophagy/autophagy via the mTOR/ULK1 pathway [38].

To investigate whether metformin activates the AMPK pathway in LHON fibroblasts, we measured phosphorylation of AMPKα1/2 at Thr183/Thr172 by immunoblotting. Representative blots show phospho‐AMPKα1/2, total AMPKα1/2, and β‐actin as a loading control in cells treated with 0, 50, and 1000 μm metformin for 24 h (Fig. 6H). Quantification of phospho‐AMPKα1/2 relative to total AMPKα1/2, normalized to β‐actin, revealed a significant increase in phosphorylation at both 50 and 1000 μm metformin compared to untreated cells (Fig. 6I). Sample‐specific results are shown in Fig. S20.

Immunofluorescence staining further revealed that metformin significantly increased AMPKβ1 (Ser182) phosphorylation in both healthy control and LHON fibroblasts compared to untreated cells (Fig. 6J). The response appeared more pronounced in healthy fibroblasts (Fig. S21), suggesting that basal mitochondrial dysfunction in LHON may limit the extent of AMPKβ1 activation. Representative images confirmed clear nuclear and cytoplasmic AMPKβ1 staining in metformin‐treated cells, whereas untreated cells showed weaker and more diffuse signals (Fig. 6K). The specificity of the signal was validated by the absence of fluorescence in experimental controls lacking primary antibody. These findings indicate that metformin enhances AMPKβ1 phosphorylation across both fibroblast groups, consistent with its role in modulating AMPK signaling and mitochondrial quality control.

The effect of metformin on mTORC1 activity was assessed by immunofluorescence staining for phospho‐p70 S6 kinase (Thr389), a downstream target of mTORC1. Quantification of fluorescence intensity revealed that metformin (50 μm, 24 h) significantly increased phospho‐p70(S6K) levels in healthy control fibroblasts compared with untreated cells (Fig. S22A). In contrast, LHON fibroblasts showed no significant change in phospho‐p70(S6K) intensity under the same treatment conditions (Fig. S22B). Representative images confirmed stronger phospho‐S6K staining in metformin‐treated healthy fibroblasts, whereas LHON fibroblasts exhibited minimal differences between treated and untreated conditions (Fig. S22C). Signal specificity was validated by experimental controls lacking the primary antibody. These results indicate that metformin enhances mTORC1 activity in healthy fibroblasts but has a limited impact on this pathway in LHON fibroblasts.

### Metformin preserved mitochondrial mass while enhancing PGC‐1α expression in healthy control and LHON fibroblasts

Mitochondrial mass is maintained by a dynamic balance between fission, fusion, biogenesis, and degradation. Excessive fission and fragmentation are typically associated with reduced mtDNA content [39], while fusion, which facilitates the formation of more tubular mitochondria, tends to increase mitochondrial mass [39, 40]. Mitophagy also contributes by selectively degrading damaged mitochondria [41]. In LHON, mtDNA mutations disrupt this balance, often shifting dynamics toward fission.

**Fig. 7:**
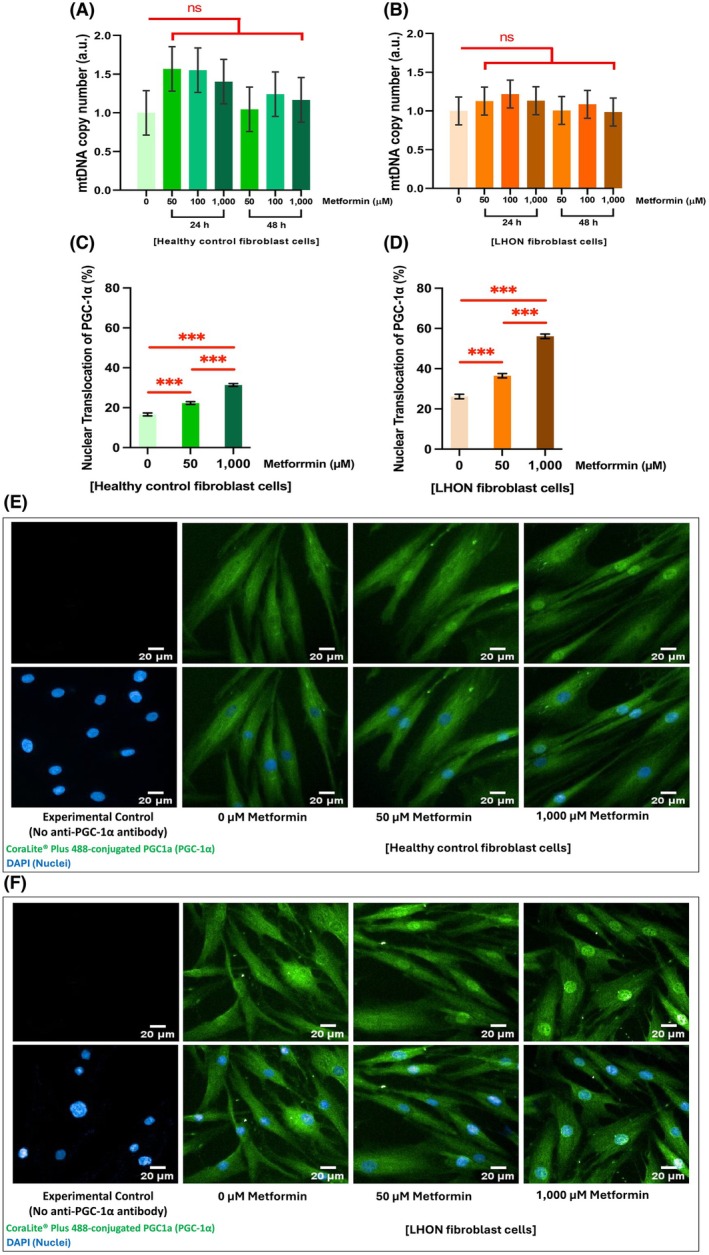
Metformin does not alter mitochondrial mass while enhancing PGC‐1α expression in healthy control and LHON fibroblasts. (A, B) Relative mtDNA copy number (normalized to nuclear DNA) shows no significant change after 24 h of metformin treatment (50 or 1000 μm), indicating stable mitochondrial mass. (C, D) Metformin significantly increases PGC‐1α nuclear translocation in both healthy control and LHON fibroblasts, consistent with activation of mitochondrial biogenesis pathways. (E, F) Representative immunofluorescence images show increased nuclear PGC‐1α (green) in metformin‐treated cells compared with untreated controls. Nuclei were counterstained with DAPI (blue). Imaging: Operetta CLS™, 20× objective; scale bar = 20 μm. Sampling: 25–45 fields per well, 2500–9000 cells per condition, in duplicate; *n* = 3 biological samples per group. Three independent experiments were performed for each sample (controls *n* = 9; LHON *n* = 11 for mtDNA quantification). Bar graphs show mean ± 95% CI. Statistical analysis by two‐way ANOVA with Tukey's post‐test; *P < 0.05, **P < 0.01, and ***P < 0.001; ns = not significant. a.u. = arbitrary units.

In this study, mtDNA copy number was used as a measure of mitochondrial mass. Metformin treatment did not significantly alter mitochondrial mass in either healthy control (Fig. 7A) or LHON fibroblast (Fig. 7B) compared with untreated cells. This suggested that the observed increase in mitophagy was not accompanied by a loss of overall mitochondrial content. However, a slight increase in mtDNA copy number was noted at 50, 100, and 1000 μm metformin after 24 h of treatment. Data for individual LHON samples are provided in Fig. S23.

Metformin significantly increased nuclear translocation of the peroxisome proliferator‐activated receptor‐gamma coactivator 1‐alpha (PGC‐1α), a reliable indicator of mitochondrial biogenesis and metabolic remodeling [42, 43], in healthy controls and LHON fibroblasts after 24 h of treatment at 50 and 1000 μm (Fig. 7C–F). Fig. S24 provides results for individual LHON samples.

These results indicate that metformin did not reduce mitochondrial mass while enhancing mitophagy and promoting mitochondrial biogenesis, consistent with a balanced modulation of mitochondrial quality control. While maintaining overall mitochondrial content, promoting the removal of damaged mitochondria, and supporting the maintenance or expansion of the healthy mitochondrial pool, metformin shows potential as a therapeutic strategy to mitigate mitochondrial dysfunction in LHON.

## Discussion

LHON is characterized by markedly reduced complex I activity in peripheral blood mononuclear cells (PBMCs) and fibroblasts [24], leading to a profound decrease in ATP production [44]. Our findings confirm this deficit; LHON fibroblasts exhibited shorter, fragmented mitochondrial networks with fewer branches, alongside reduced ATP and MMP compared with healthy controls (Fig. 1). This imbalance in mitochondrial dynamics, favoring fission, contributes to neurodegeneration and LHON pathology [45]. Excessive mitochondrial fission is strongly linked to increased ROS production and oxidative stress [6], which in turn accelerates cellular injury, as demonstrated in a mouse model of LHON [7].

Metformin demonstrated measurable improvements under these conditions. Treatment with 50 μm for 24 h activated autophagy in LHON fibroblasts, evidenced by LC3BI to LC3BII conversion. Moreover, LC3B puncta analysis further confirmed that metformin increased LC3B intensity, and additional accumulation in the presence of chloroquine demonstrated that these effects reflected enhanced autophagic flux rather than a block in degradation (Fig. 6F,G). Mitophagy analysis also showed a dose‐dependent increase, beginning as low as 5 μm, without a reduction of mitochondrial mass or increased cell death (Figs S25 and S26). Prior studies have shown that metformin enhances lysosomal acidity and degradative activity [46], increases autophagic flux in fibroblasts [47]. By stimulating AMPK/ULK1‐mediated autophagy, metformin has been reported to mitigate ROS accumulation, stabilize MMP, and prevent apoptosis in primary human retinal pigment epithelial cells [48]. These findings indicate that metformin enhances mitochondrial quality by promoting ongoing autophagy and mitophagy.

Metformin also influenced mitochondrial biogenesis. We observed an increase in PGC‐1α nuclear localization following metformin treatment, both in healthy controls and in LHON fibroblasts (Fig. 7C–F). Since the transcriptional activity of PGC‐1α requires nuclear presence, these findings provide functional evidence for metformin‐induced biogenesis, complementing the observed mitophagy. Similar effects of metformin on PGC‐1α activation and mitochondrial biogenesis have been reported in fibroblasts from patients with Down syndrome [15] and in mouse brown adipocytes [49]. Together with our results, these findings suggest that metformin supports a coordinated response, coupling enhanced mitochondrial clearance with biogenesis to maintain homeostasis.

AMPK signaling was further confirmed by two complementary approaches. Immunoblotting revealed increased phosphorylation of AMPKα1/2 at Thr183/Thr172 in LHON fibroblasts after metformin treatment (Fig. 6H,I). In addition, immunofluorescence showed significant increases in AMPKβ1 (Ser182) phosphorylation in both healthy control and LHON fibroblasts (Fig. 6J,K). While phosphorylation of β‐subunits does not directly reflect catalytic activity, the consistent increase alongside AMPKα activation suggests coordinated regulation within the holoenzyme complex. Interestingly, the response was more pronounced in healthy fibroblasts, raising the possibility that baseline mitochondrial dysfunction in LHON may constrain the extent of AMPKβ1 activation.

Mitochondrial respiration assays further demonstrated that metformin at clinically relevant concentrations (50–100 μm) did not impair oxidative phosphorylation. In fact, basal and ATP‐linked respiration increased in some samples. This is consistent with previous evidence showing that complex I inhibition occurs only at suprapharmacological concentrations (hundreds of μm to mm) [50], whereas therapeutic plasma concentrations of metformin do not impair complex I activity and may, in fact, enhance respiration [51]. Even at higher concentrations, metformin causes only minimal inhibition of NADH oxidation in submitochondrial particles, indicating that it does not directly interfere with NADH oxidation [52]. Clinical observations support this interpretation. Larsen, *et al*. [53] reported no complex I inhibition in skeletal muscle biopsies from diabetic patients treating with 1000–2000 mg metformin daily. Collectively, these results argue that therapeutic concentrations of metformin support rather than impair mitochondrial function in LHON fibroblasts.

Metformin also influenced mitochondrial morphology. Concentrations between 5 and 100 μm consistently reduced fragmentation and promoted an elongated mitochondrial network under basal conditions, while 10–100 μm attenuated fragmentation under phenanthroline‐induced stress. In L2 LHON fibroblasts, 50 μm metformin significantly decreased the p‐DRP1/total DRP1 ratio, suggesting reduced fission activity. These findings align with prior reports showing that metformin reduces DRP1 and FIS1 expression in leukocytes of diabetic patients [54] and reduces fragmentation through AMPK‐dependent mechanisms in neuronal [13] and metabolic models [55]. Rather than restoring networks to wild‐type, these findings indicate that metformin shifts the balance between fission and fusion toward a more interconnected state.

The concentrations used in our experiments correspond closely to clinically observed plasma levels. In healthy individuals, a single 1000 mg oral dose produces peak plasma levels of 25 μm within 3 h, with steady‐state concentrations of 5–10 μm [56]. Comparable therapeutic plasma levels ranging from 10 to 40 μm have also been independently reported [57]. At clinically observed exposures (~40–70 μm in the portal vein; ~10–40 μm systemic), metformin acts mainly through signaling and gut‐axis pathways rather than direct mitochondrial inhibition [58, 59]. At these low doses, metformin activates AMPK via a lysosomal PEN2‐ATP6AP1/v‐ATPase mechanism [16] and suppresses hepatic gluconeogenesis through glucagon/cAMP axis‐mediated inhibition of adenylate cyclase and AMPK‐dependent activation of PDE4B [60, 61]. Intestinal mechanisms also contribute at therapeutic levels, including stimulation of GLP‐1 secretion [62] and modulation of the microbiome‐bile acid‐FXR pathway, as illustrated by GUDCA‐linked FXR antagonism [63].

In contrast, millimolar concentrations—standard in many *in vitro* studies—impose bioenergetic stress by directly inhibiting the respiratory chain, most consistently complex I [50, 64], with recent work suggesting possible effects on complex IV that remain unresolved [65]. In our study, the effective range of 10–100 μm enhanced ATP production, stabilized membrane potential, and promoted mitophagy and AMPK activation in LHON fibroblasts, consistent with clinically relevant exposures and distinct from the nonphysiological stress observed at suprapharmacological doses.

Metformin is transported into cells via OCTs. A study on ocular membrane transporter localization reported the presence of hOCT1 (*SLC22A1*), hOCT2 (*SLC22A2*), and hOCT3 (*SLC22A3*) transcripts in the human retina [66], though robust evidence for protein expression in the neuronal retina remains limited. hOCT1 and hOCT2 transcripts have also been detected in human retinal pigment epithelial (RPE) cells, including D407 and ARPE‐19. In addition, OCT3 is expressed in mouse RPE and across multiple neural retinal cell types, including photoreceptors, interneurons, and RGCs [67, 68]. Importantly, there is no evidence that OCT expression is lost in retinal pathologies, including LHON. These data support the hypothesis that RGCs likely retain the ability to take up metformin via OCTs, providing a plausible mechanistic basis for the therapeutic effects we observed in fibroblasts.

Currently, idebenone is the only approved pharmacological treatment for LHON. As a short‐chain benzoquinone, idebenone acts as a mitochondrial electron carrier, bypassing complex I dysfunction by transferring electrons directly to complex III, thereby restoring ATP synthesis and mitigating oxidative stress [69, 70]. Clinical trials have substantiated its benefit in visual recovery, especially when administered early in disease progression [71, 72]. Metformin, by contrast, exerts its effects indirectly through AMPK activation, enhancing mitochondrial quality control via autophagy and mitophagy, while also reducing both ROSs and inflammatory responses [12, 73]. Though clinical trials in LHON have not yet been conducted, preclinical studies highlight metformin's neuroprotective effects in models of mitochondrial dysfunction and optic nerve injury [74, 75].

Both approaches possess inherent limitations. Idebenone requires early administration to achieve efficacy [70], while metformin, by contrast, carries a theoretical risk of lactic acidosis in patients with severe mitochondrial dysfunction. However, this risk is very low with standard monitoring with an incidence of ~5 cases per 100 000 patient‐years [76].

**Fig. 8:**
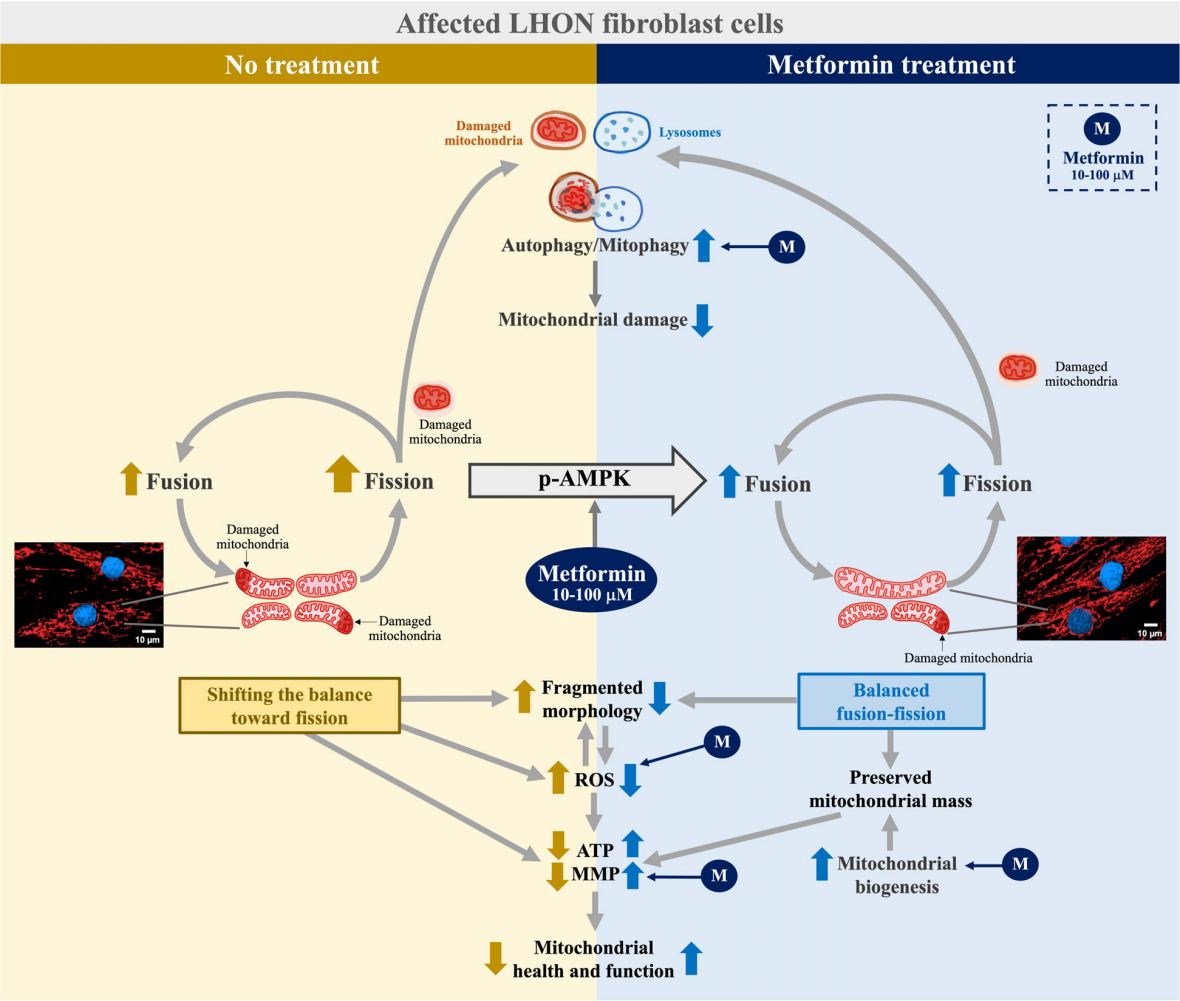
Proposed model of metformin‐mediated mitochondrial quality‐control enhancement in LHON fibroblasts. Metformin activates AMPK signaling, shifting mitochondrial dynamics toward reduced fission and enhanced fusion. At optimal concentrations (10–100 μm), it maintains mitochondrial mass, stimulates autophagy and mitophagy, and promotes PGC‐1α nuclear translocation and biogenesis. These coordinated effects lower mitochondrial ROS, elevate ATP, and improve membrane potential, supporting mitochondrial health in LHON fibroblasts.

In summary, our results show that metformin at clinically relevant concentrations modulates mitochondrial morphology, enhances mitophagy and autophagy, stabilizes MMP, reduces ROS, increases PGC‐1α, and maintains mitochondrial mass in LHON fibroblasts (Fig. 8). These changes occur without impairing oxidative phosphorylation and are associated with AMPK activation through both α and β subunits. Given its affordability, safety, and widespread clinical use, metformin represents a promising candidate for repurposing in LHON. Further studies are warranted to evaluate its efficacy, determine its ability to reach RGCs at therapeutic concentrations, and assess its impact on vision preservation in patients.

## Conflict of interest

The authors declare no conflict of interest.

## Author contributions

Both CPa and CPe contributed to the manuscript preparation. CPe composed grants and conceived and organized experiments. CPa, RS, and MT conducted the experiments. CPe contributed to the development and execution of the infrastructure. All authors provided critical feedback and aided in the development of the manuscript.

## Supporting information


**Table S1.** Sample information for LHON patients and healthy volunteers.
**Table S2.** Primer sequences for mtDNA and nuclear DNA qPCR.
**Table S3.** Measurement of autophagic flux.
**Fig. S1.** CellProfiler 4.0.7 pipeline for mitophagy quantification.
**Fig. S2.** *MT‐ND4* m.11778 electropherograms: LHON vs healthy controls.
**Fig. S3.** *MT‐ND1* m.3460 electropherograms in LHON fibroblasts.
**Fig. S4.** *MT‐ND1* m.3460 electropherograms in healthy controls.
**Fig. S5.** *MT‐ND6* m.14484 electropherograms in LHON fibroblasts.
**Fig. S6.** *MT‐ND6* m.14484 electropherograms in healthy controls.
**Fig. S7.** Quantitative analysis of mitochondrial morphology: LHON vs healthy controls.
**Fig. S8.** Metformin reduces fragmentation and increases mitochondrial length in LHON fibroblasts (24 h).
**Fig. S9.** Metformin attenuates phenanthroline‐induced fragmentation and increases mitochondrial length in LHON fibroblasts.
**Fig. S10.** DRP1 activation decreases in a subset of LHON samples following metformin treatment.
**Fig. S11.** Metformin enhances ATP generation in specific LHON fibroblast samples.
**Fig. S12.** Metformin increases mitochondrial membrane potential (MMP) in LHON fibroblasts.
**Fig. S13.** Seahorse oxygen consumption rate (OCR) traces of LHON fibroblasts.
**Fig. S14.** Respiratory parameters in L1 fibroblasts treated with metformin.
**Fig. S15.** Respiratory parameters in L2 fibroblasts treated with metformin.
**Fig. S16.** Respiratory parameters in L3 fibroblasts treated with metformin.
**Fig. S17.** Metformin alleviates H_2_O_2_‐induced mitochondrial oxidative stress in LHON fibroblasts.
**Fig. S18.** Metformin enhances mitophagy in LHON fibroblasts.
**Fig. S19.** Metformin upregulates autophagic proteins in LHON fibroblasts.
**Fig. S20.** Metformin increases AMPK activation in LHON fibroblasts.
**Fig. S21.** pAMPKβ1 levels are lower in LHON fibroblasts compared with healthy controls.
**Fig. S22.** Metformin increases phospho‐p70(S6K) (Thr389) in healthy control fibroblasts but not in LHON fibroblasts.
**Fig. S23.** Metformin does not alter mitochondrial mass in LHON fibroblasts.
**Fig. S24.** Metformin induces PGC‐1α nuclear translocation in specific LHON fibroblast samples.
**Fig. S25.** Cytotoxicity imaging in fibroblasts treated with metformin.
**Fig. S26.** Quantitative analysis of cytoxicity in LHON fibroblasts following metformin treatment.

## Data Availability

The data that support the findings of this study are available in the Supporting Information section.
