# Supplementary material for: Metformin promotes mitochondrial integrity through AMPK‐signaling in Leber's hereditary optic neuropathy
Source: FEBS Open Bio. 2025 Nov 23;16(5):885–905. doi: 10.1002/2211-5463.70165 (PMC13145345; doi:10.1002/2211-5463.70165)
Supplement: Supplementary file 1 — Table S1. Sample information for LHON patients and healthy volunteers. Table S2. Primer sequences for mtDNA and nuclear DNA qPCR. Table S3. Measurement of autophagic flux. Fig. S1. CellProfiler 4.0.7 pipeline for mitophagy quantification. Fig. S2. MT‐ND4 m.11778 electropherograms: LHON vs healthy controls. Fig. S3. MT‐ND1 m.3460 electropherograms in LHON fibroblasts. Fig. S4. MT‐ND1 m.3460 electropherograms in healthy controls. Fig. S5. MT‐ND6 m.14484 electropherograms in LHON fibroblasts. Fig. S6. MT‐ND6 m.14484 electropherograms in healthy controls. Fig. S7. Quantitative analysis of mitochondrial morphology: LHON vs healthy controls. Fig. S8. Metformin reduces fragmentation and increases mitochondrial length in LHON fibroblasts (24 h). Fig. S9. Metformin attenuates phenanthroline‐induced fragmentation and increases mitochondrial length in LHON fibroblasts. Fig. S10. DRP1 activation decreases in a subset of LHON samples following metformin treatment. Fig. S11. Metformin enhances ATP generation in specific LHON fibroblast samples. Fig. S12. Metformin increases mitochondrial membrane potential (MMP) in LHON fibroblasts. Fig. S13. Seahorse oxygen consumption rate (OCR) traces of LHON fibroblasts. Fig. S14. Respiratory parameters in L1 fibroblasts treated with metformin. Fig. S15. Respiratory parameters in L2 fibroblasts treated with metformin. Fig. S16. Respiratory parameters in L3 fibroblasts treated with metformin. Fig. S17. Metformin alleviates H2O2‐induced mitochondrial oxidative stress in LHON fibroblasts. Fig. S18. Metformin enhances mitophagy in LHON fibroblasts. Fig. S19. Metformin upregulates autophagic proteins in LHON fibroblasts. Fig. S20. Metformin increases AMPK activation in LHON fibroblasts. Fig. S21. pAMPKβ1 levels are lower in LHON fibroblasts compared with healthy controls. Fig. S22. Metformin increases phospho‐p70(S6K) (Thr389) in healthy control fibroblasts but not in LHON fibroblasts. Fig. S23. Metformin does not alter mitochondrial mass in LHON [file FEB4-16-885-s001.docx]

**Supporting Information**

**Metformin promotes mitochondrial integrity through AMPK-signaling in Leber’s hereditary optic neuropathy**

Chatnapa Panusatid, Rapasviranda Soiyangsuk, Maneeluck Tanadjindarat, Chayanon Peerapittayamongkol*

Department of Biochemistry, Faculty of Medicine Siriraj Hospital, Mahidol University, Bangkok, Thailand

*Corresponding author: Chayanon Peerapittayamongkol

Corresponding Author’s email: chayanon.pee@mahidol.ac.th

**Supplemental Table S1.** **Sample information for LHON patients and healthy volunteers**

| **Sample ID** | **Skin Biopsy Source** | **Gender** | **Age at Recruitment (years)** | **Age of Onset**  **(years)** | **Position of Mitochondrial Mutation** |
| --- | --- | --- | --- | --- | --- |
| L1 | Affected patient | Male | 25 | 15 | m.11778, no mutation at m.3460 and m.14484 |
| L2 | Affected patient | Male | 31 | 15 | m.11778, no mutation at m.3460 and m.14484 |
| L3 | Affected patient | Male | 14 | 7 | m.11778, no mutation at m.3460 and m.14484 |
| L4 | Affected patient | Female | 35 | 10 | m.11778, no mutation at m.3460 and m.14484 |
| H1 | Healthy volunteer | Male | 37 | - | No mutation at m.11778, m.3460, and m.14484 |
| H2 | Healthy volunteer | Male | 35 | - | No mutation at m.11778, m.3460, and m.14484 |
| H3 | Healthy volunteer | Male | 34 | - | No mutation at m.11778, m.3460, and m.14484 |

**Supplemental Table S2.** **Primer sequences for mtDNA and nuclear DNA qPCR**

| **Target genes** | **Primer sequences** | **Supplier** |
| --- | --- | --- |
| *MT-TL1* (mtDNA) | Forward primer: 5’- CACCCAAGAACAGGGTTTGT -3’  Reverse primer: 5’- TGGCCATGGGTATGTTGTTA -3’ | Sigma, USA |
| *ZHX2* (nuclear DNA) | Forward primer: 5’- CTTCTGTACAGCTGCCACCA -3’  Reverse primer: 5’- CTGTCAGCCAGGACAACTCA -3’ | Sigma, USA |

**Supplemental Table S3.** **Measurement of autophagic flux**

| **Treatment Conditions** | **Mean LC3B Fluorescence Intensity** | **Autophagic Flux*** |
| --- | --- | --- |
| Untreated cells | 112.8 | 143.6  (Untreated) |
| Untreated cells + 50 µM Chloroquine | 256.5 |  |
| 50 µM Metformin | 114.6 | 162.5  (50 µM Metformin) |
| 50 µM Metformin + 50 µM Chloroquine | 277.1 |  |

*Autophagic flux was calculated using the formula:

Autophagic flux = LC3B (with lysosomal inhibitor) - LC3B (without inhibitor)

**A pipeline for the quantification of mitophagy levels by CellProfiler program**

To analyze mitophagy, the fluorescent cell images captured by the Operetta CLS^TM^ underwent processing using the Metadata extraction and Illumination correction pipeline, as previously described in our published work [1]. Subsequently, the stained nuclei, marked by the Hoechst 33342 dye, were used to locate the cells. The MITO-ID® Red channel was then utilized to create a secondary object that outlines the cell boundary and cytoplasm (Figure S1.A). Lysosomes were identified by analyzing the LysoGreen channel (Figure S1.B). The analysis of mitochondria was conducted on the MITO-ID® Red channels, and subsequently, the identified mitochondria and lysosomes were reduced to their skeletal forms (Figure S1.A and S1.B). The mitochondrial skeletons were linked to the cytoplasm, after which the lysosomal skeletons were related to the mitochondrial skeleton (Figure S1.B). The colocalized mitochondrial and lysosomal skeletons associated with the cells were then classified (Figure S1.C). Lastly, the amount of mitophagy was measured by counting how often the mitochondrial and lysosomal skeletons were in the same location. This number was then normalized against the total number of mitochondrial skeletons [2]. The detailed method for analyzing mitophagy will be published in a separate publication.


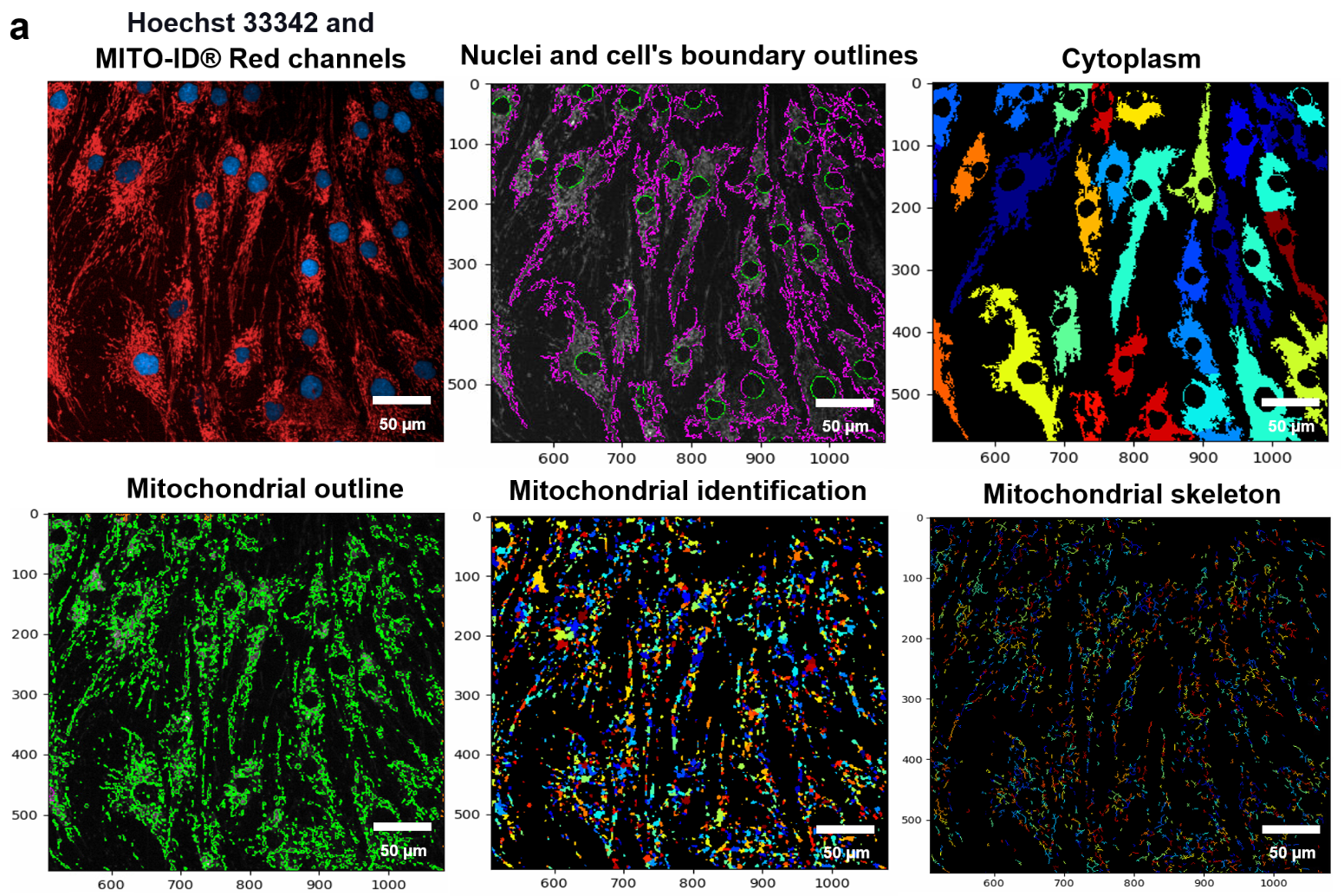


**A**


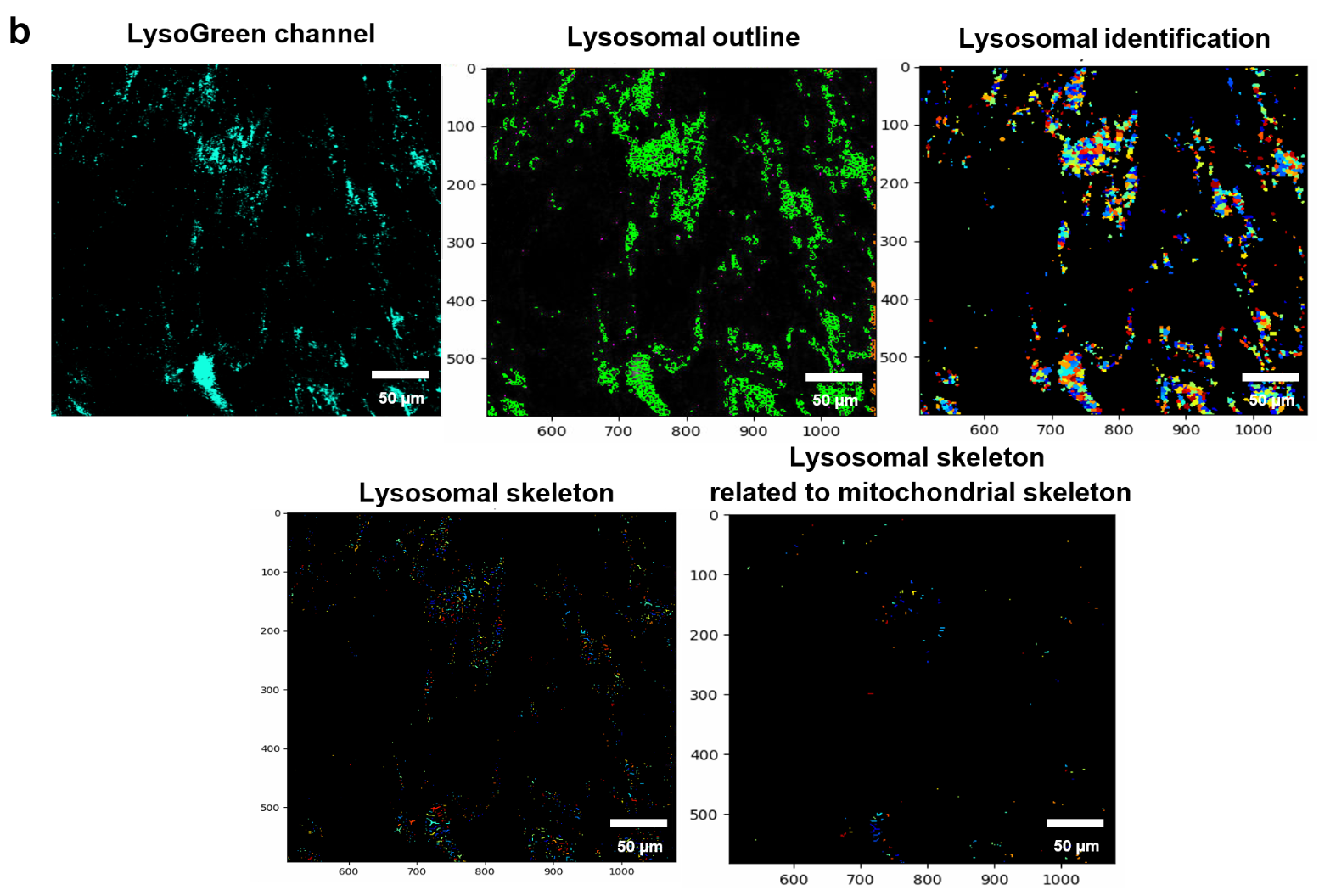


**B**


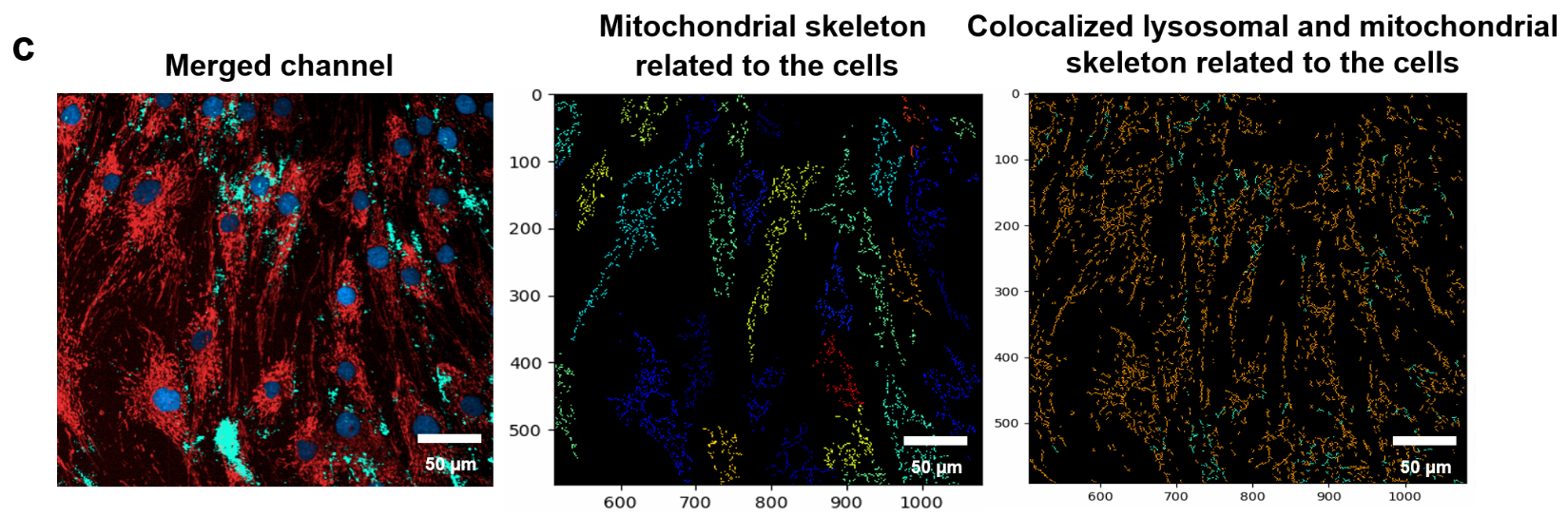


**C**

**Supplemental Figure S1. CellProfiler 4.0.7 pipeline for mitophagy quantification.** (A) Nucleus/cell boundary; (Upper panel) segmentation of nuclei (Hoechst 33342, MITO-ID® Red); cytoplasm mask defined. (Lower panel) Mitochondrial identification and skeletonization. (B) Lysosome detection; (Upper panel) Lysosome identification (LysoGreen). (Lower panel) skeletonized and association with mitochondrial skeletons. (C) Organelle colocalization; (Middle panel) mitochondrial skeletons, associated to cytoplasm. (Right panel) mito-lyso colocalization per cell. Mitophagy = colocalized mito-lyso skeleton length/total mitochondrial skeleton length. Scale bars, 50 µm.

**Image analysis using CellProfiler for PGC-1α, p70(S6K), pAMPK β1, and LC3B expression measurement**

Fluorescent images were analyzed using CellProfiler to quantify protein localization and puncta formation. Nuclei were segmented from the DAPI channel and used to define single-cell regions. The Alexa488 channel, corresponding to the target antibody, was masked by the cell boundary and partitioned into nuclear and cytoplasmic regions. Mean fluorescence intensity in the cytoplasmic compartment was measured for LC3B, pAMPK β1, and p70(S6K). For LC3B puncta analysis, the EnhanceEdges module was applied to highlight punctate structures, followed by segmentation with a carefully selected lower-bound threshold to minimize false-positive counts. For PGC-1α, staining showed a clear distinction between nuclear and cytoplasmic signals. Thresholds were applied consistently across all images acquired within the same experiment, allowing nuclei to be classified as either containing significant PGC-1α nuclear signal or not. This provided a single-cell readout of PGC-1α nuclear translocation.


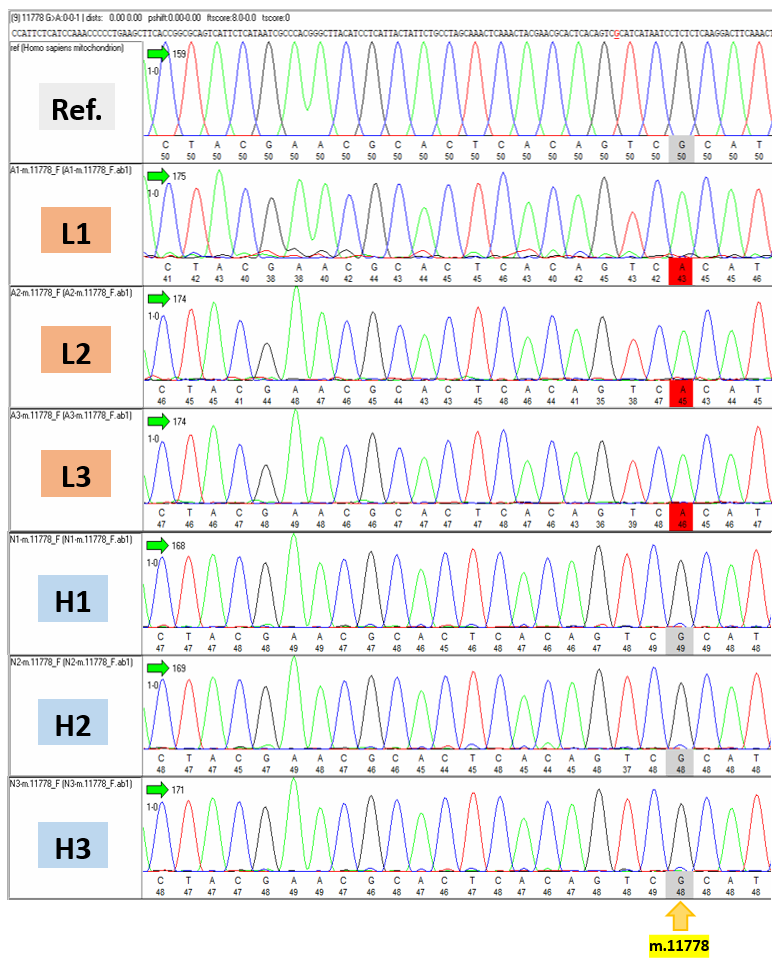


**Supplemental Figure S2. *MT-ND4* m.11778 electropherograms: LHON vs healthy controls.** Reference shows G at m.11778; LHON (L1-L3) show A; healthy (H1-H3) match reference (G).

**A**


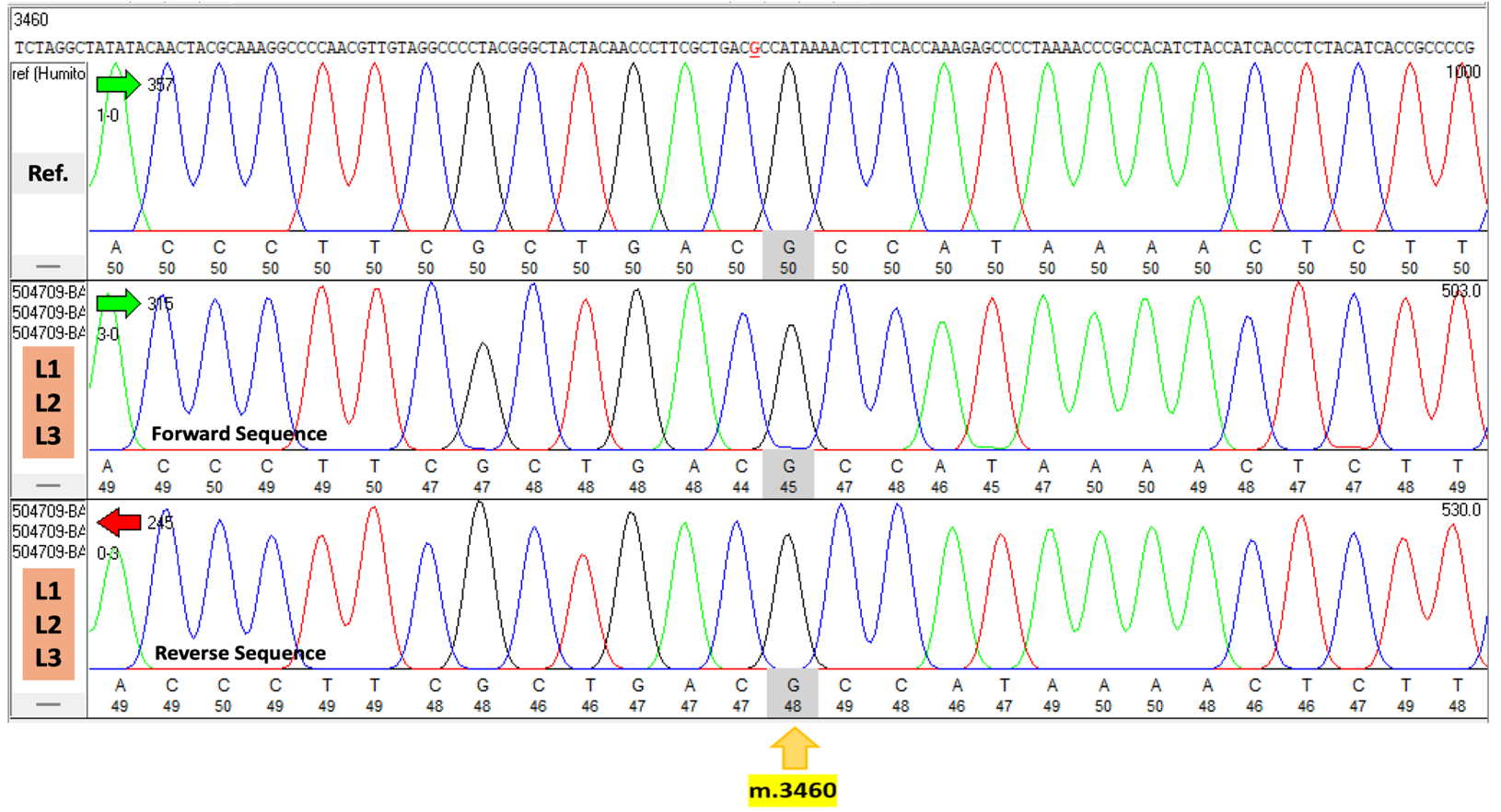


**B**


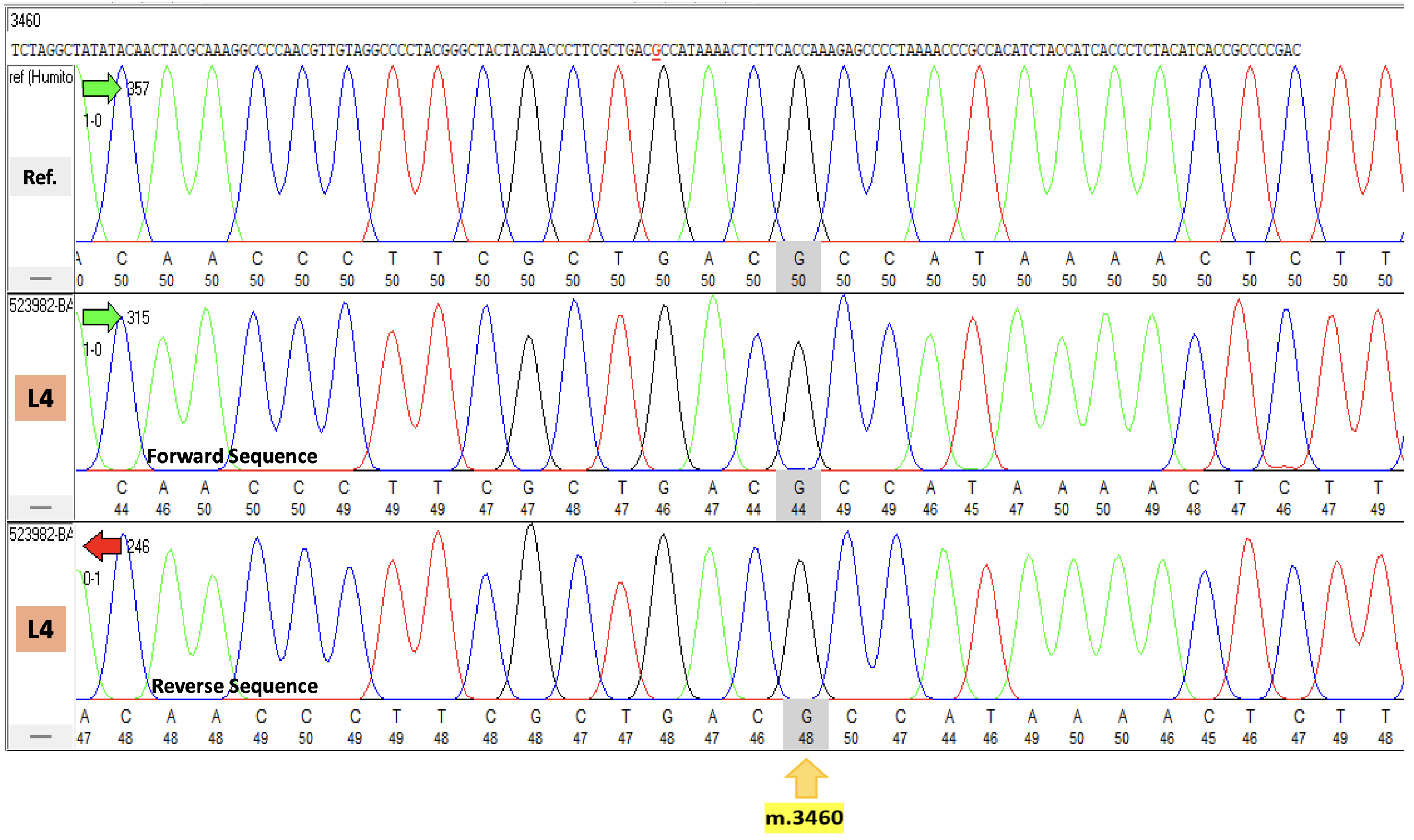


**Supplemental Figure S3. *MT-ND1* m.3460 electropherograms in LHON fibroblasts.** (A-B) Reference G at m.3460; LHON (L1-L4) forward/reverse sequences match G.


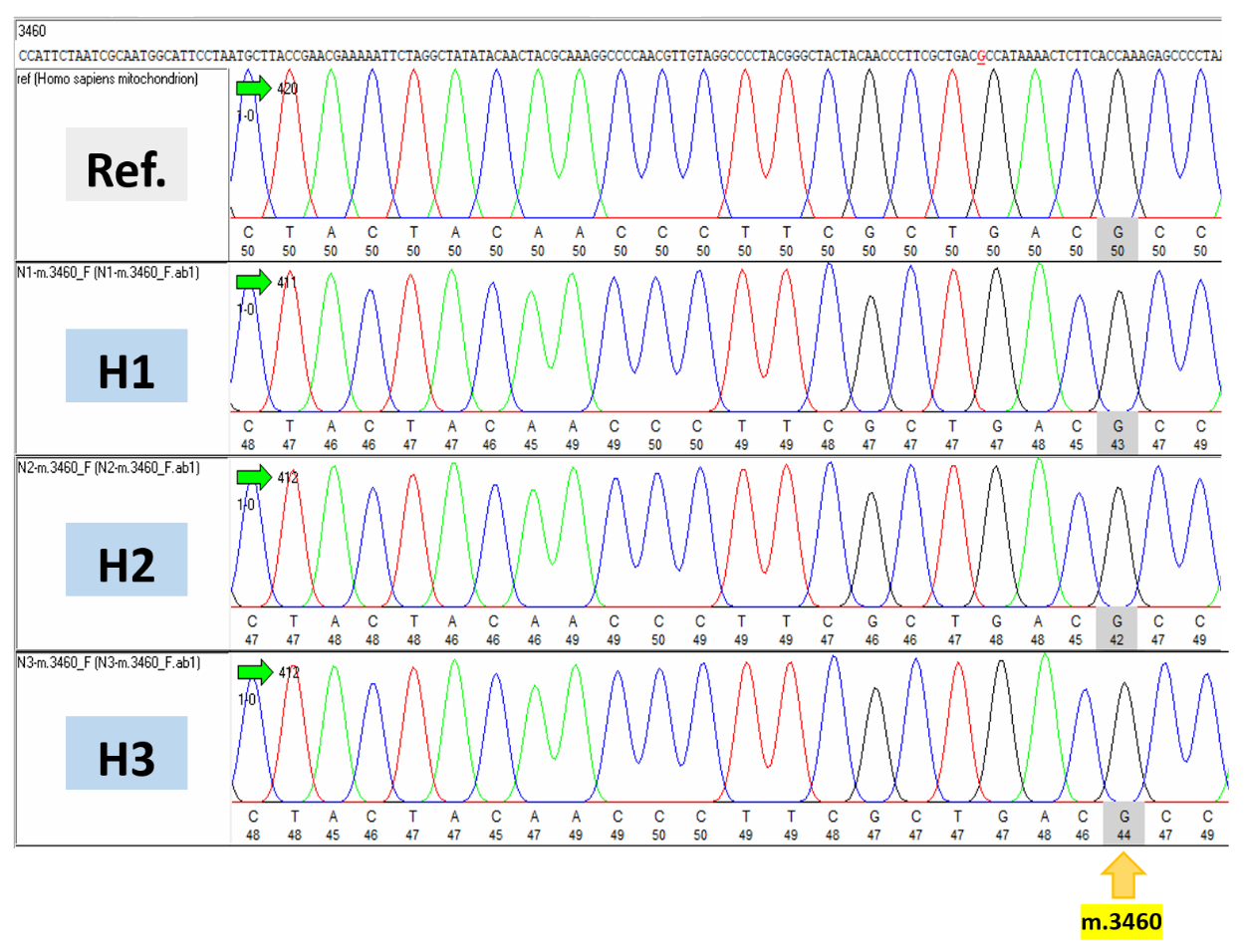


**Supplemental Figure S4. *MT-ND1* m.3460 electropherograms in healthy controls.** Reference G at m.3460; healthy (H1-H3) match reference.

**A**


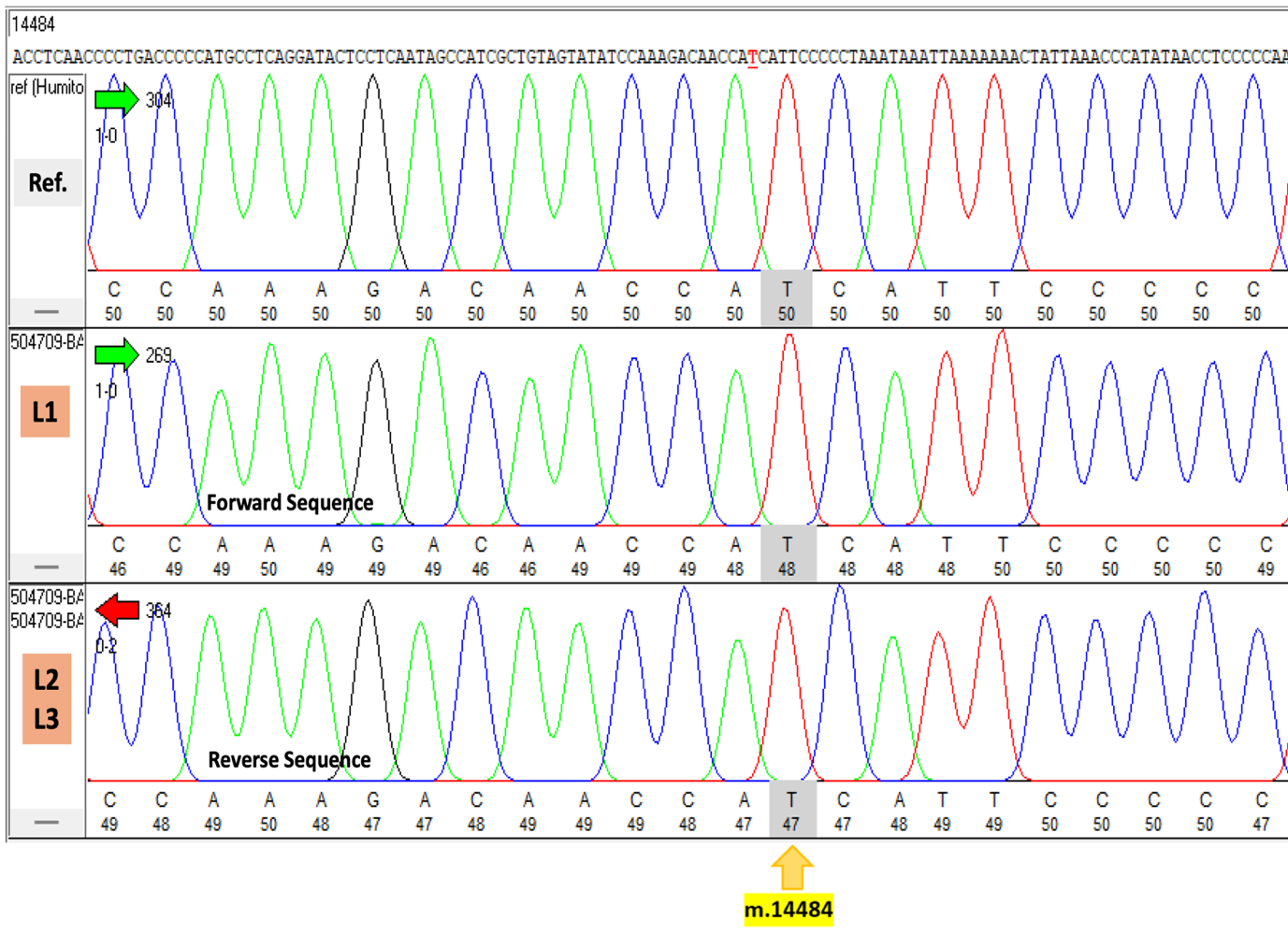


**B**


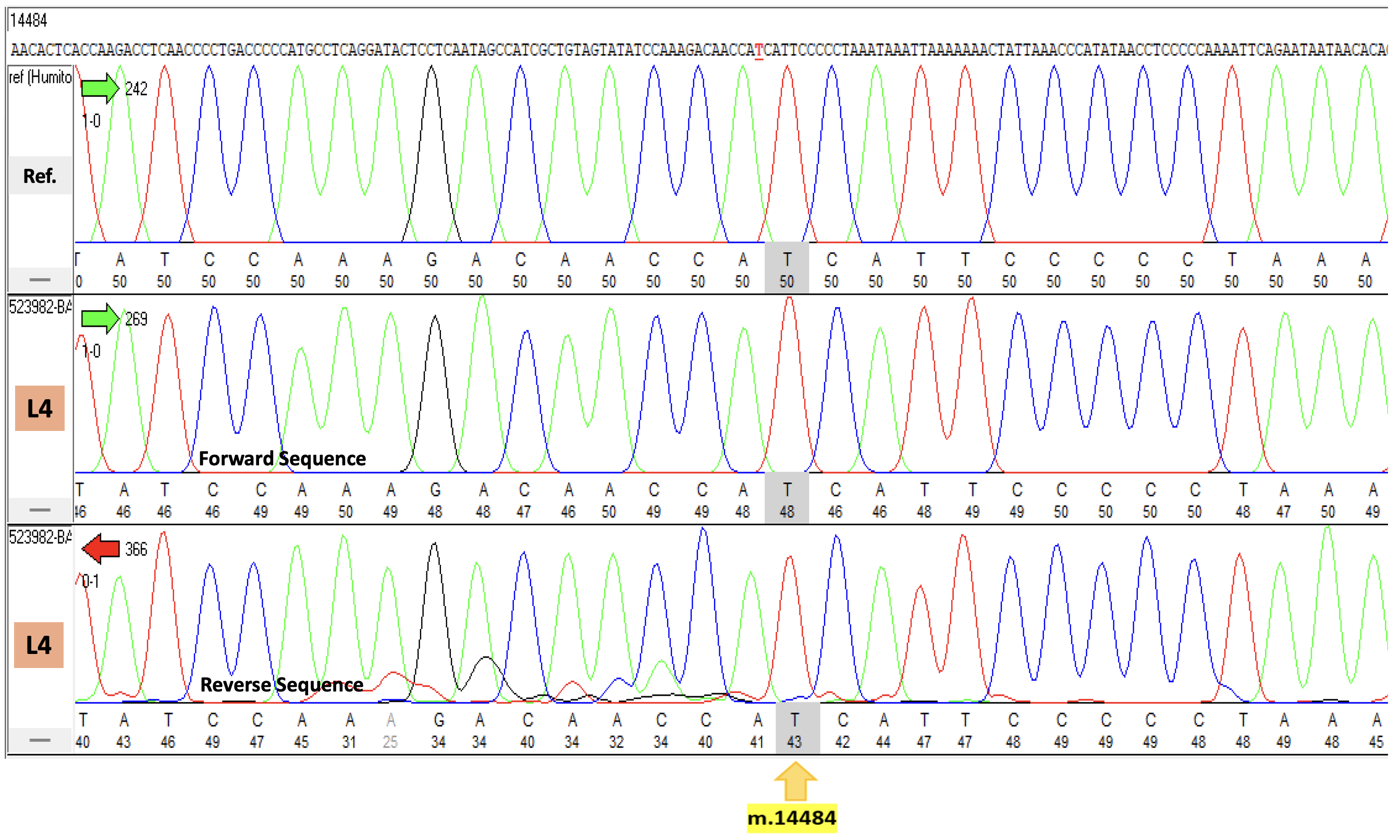


**Supplemental Figure S5. *MT-ND6* m.14484 electropherograms in LHON fibroblasts.** (A-B) Reference T at m.14484; LHON (L1-L4) forward/reverse sequences match T.

**
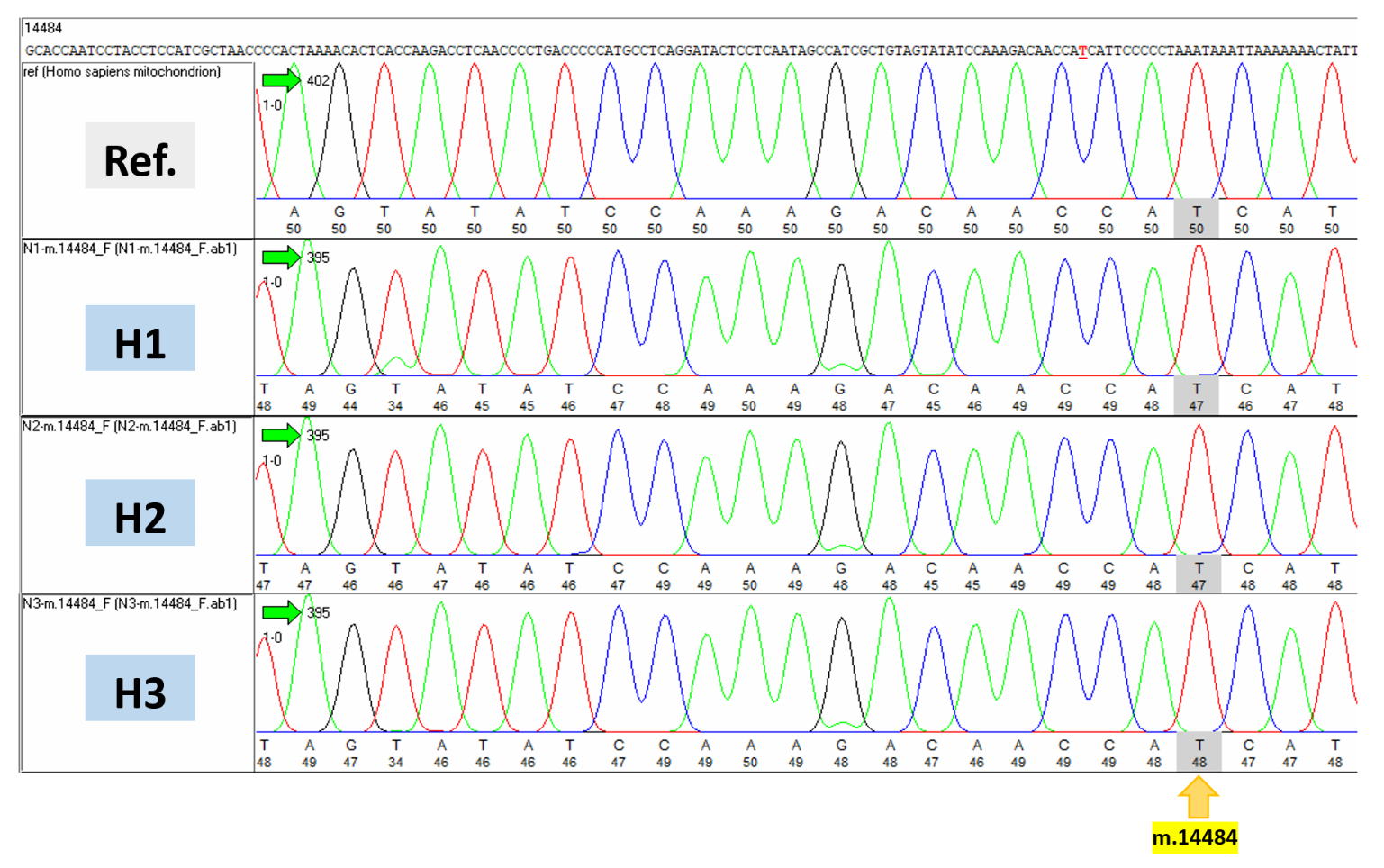
**

**Supplemental Figure S6. *MT-ND6* m.14484 electropherograms in healthy controls.** Reference T at m.14484; healthy (H1-H3) match reference.

**A B**

**** ****

**Supplemental Figure S7. Quantitative analysis of mitochondrial morphology: LHON vs healthy controls.** (A) LHON shows higher fragmentation (%) and (B) shorter length (pixels/cells). Imaging and sampling as in Figure 1. Bar graphs: mean with 95% CI (3 controls, 3 LHON). Statistics: Student’s t-test; **p* < 0.05, ***p* < 0.01, ****p* < 0.001.


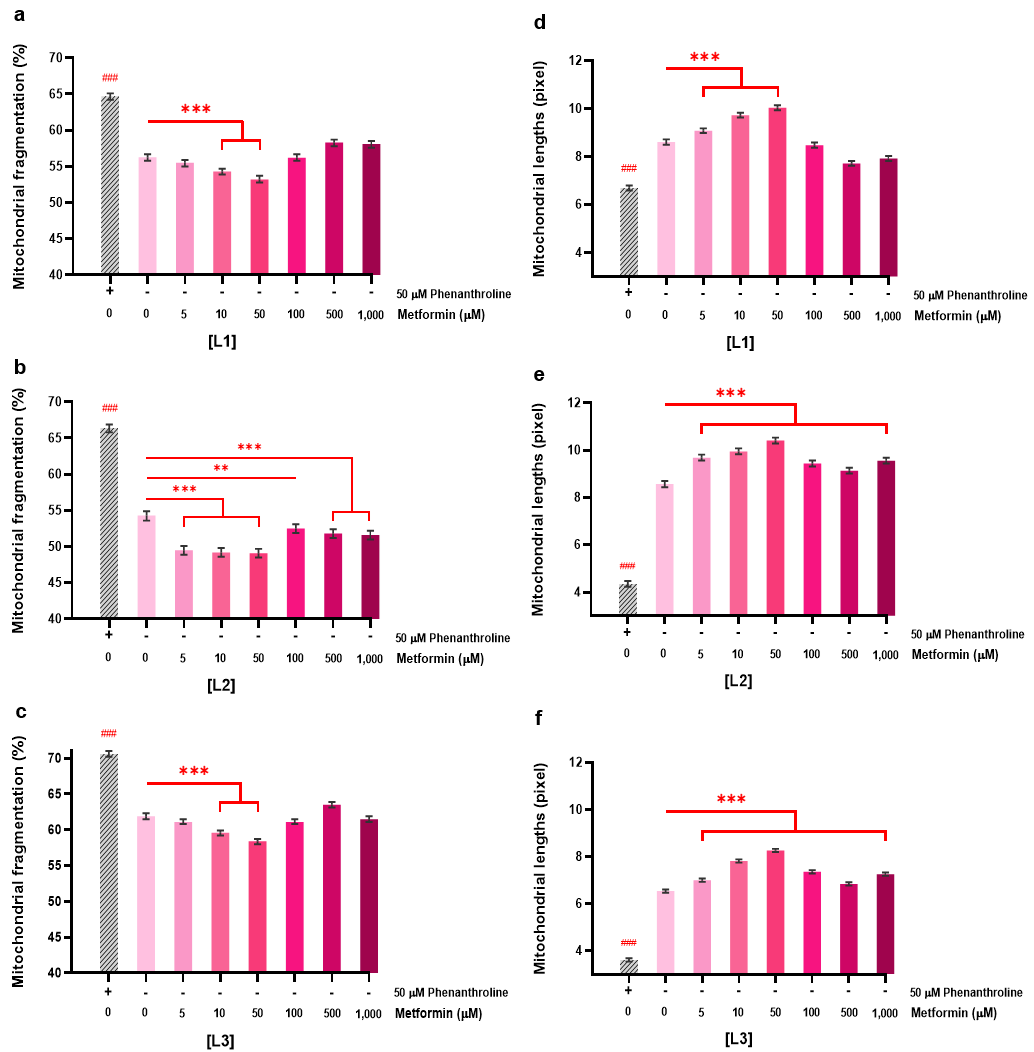


**F**

**C**

**B**

**E**

**D**

**A**

**Supplemental Figure S8. Metformin reduces fragmentation and increases mitochondrial length in LHON fibroblasts (24 h).** Left: fragmentation decreases at (A) 10-50 µM (L1), (B) 5-1,000 µM (L2), and (C) 10-50 µM (L3). Right: length increases at (D) 5-50 µM (L1), (E-F) 5-1,000 µM (L2 and L3). Design: 3 independent experiments/sample; duplicate wells; 60-80 fields/well; 1,800-6,400 cells/condition/experiment. Statistics: one-way ANOVA/Tukey; **p* < 0.05, ***p* < 0.01, ****p* < 0.001, ### phenanthroline vs untreated.


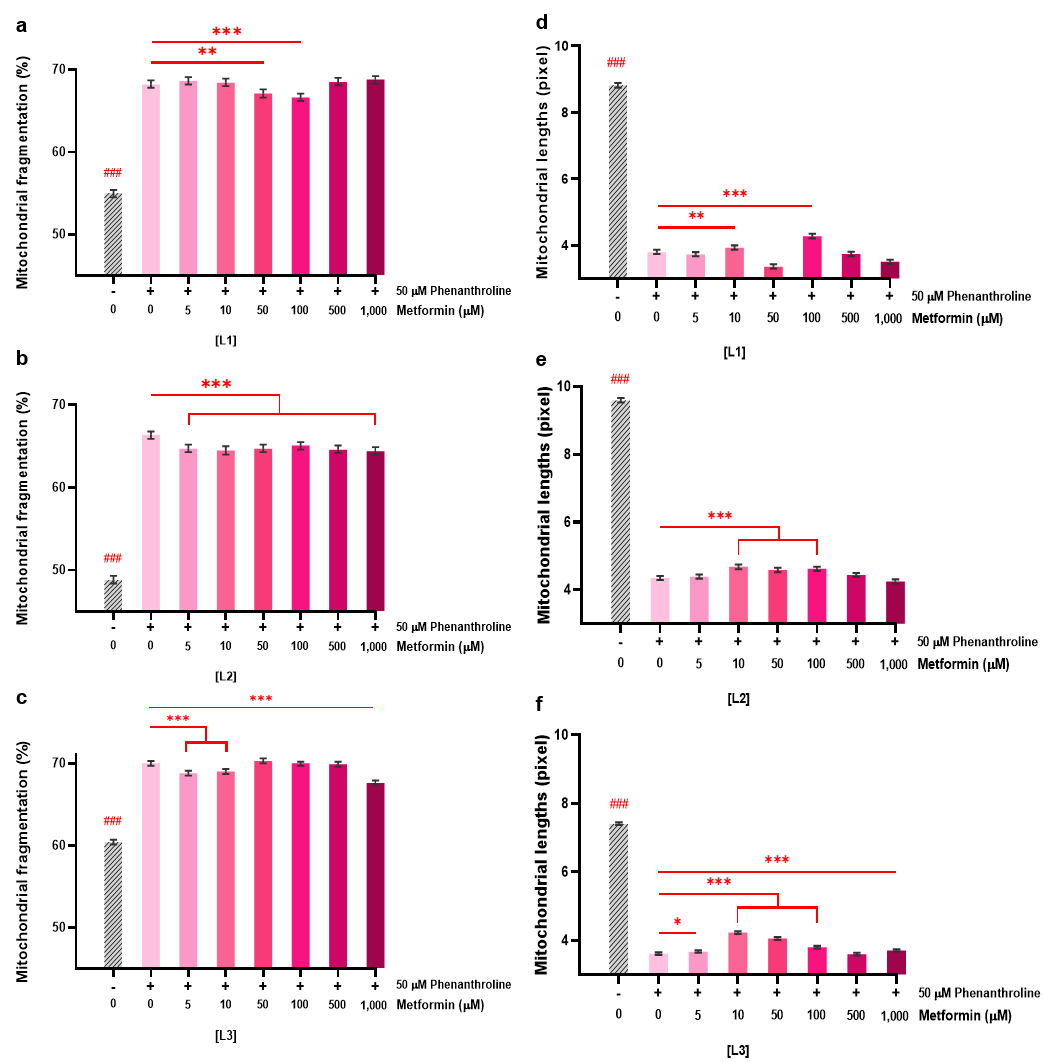


**F**

**E**

**D**

**A**

**B**

**C**

**Supplemental Figure S9.** **Metformin attenuates phenanthroline-induced fragmentation and increases mitochondrial length in LHON fibroblasts.** Left: fragmentation decreases vs phenanthroline alone at (A) 50-100 µM (L1), (B) 5-1,000 µM (L2), (C) 5-10 and 1,000 µM (L3). Right: length increases at (D) 10 and 100 µM (L1), (E) 10-100 µM (L2), (F) 5-100 and 1,000 µM (L3). Design as in S8. Statistics: ANOVA/Tukey; **p* < 0.05, ***p* < 0.01, ****p* < 0.001, ### vehicle control vs phenanthroline.


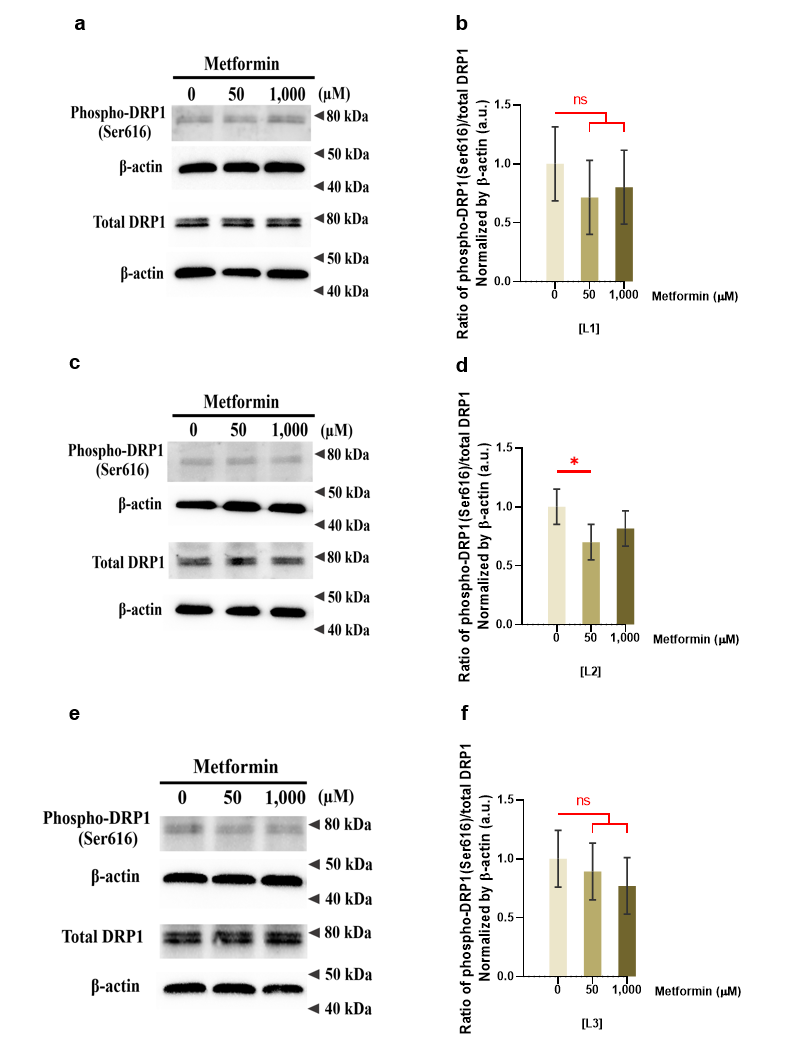


**E**

**F**

**D**

**C**

**A**

**B**

**Supplemental Figure S10. DRP1 activation decreases in a subset of LHON samples following metformin treatment.** Left: immunoblots (p-DRP1 Ser616, total DRP1, β-actin) in L1-L3 after 50 and 1,000 µM (24 h). Right: p-DRP1/DRP1 normalized to β-actin, relative to untreated (1.0). ≥3 independent experiments/sample (total = 12 experiments). Bars: mean ± 95% CI. Statistics: one-way ANOVA followed by Tukey’s test; **p* < 0.05, ***p* < 0.01, ****p* < 0.001, ns = not significant, a.u.= arbitrary units.


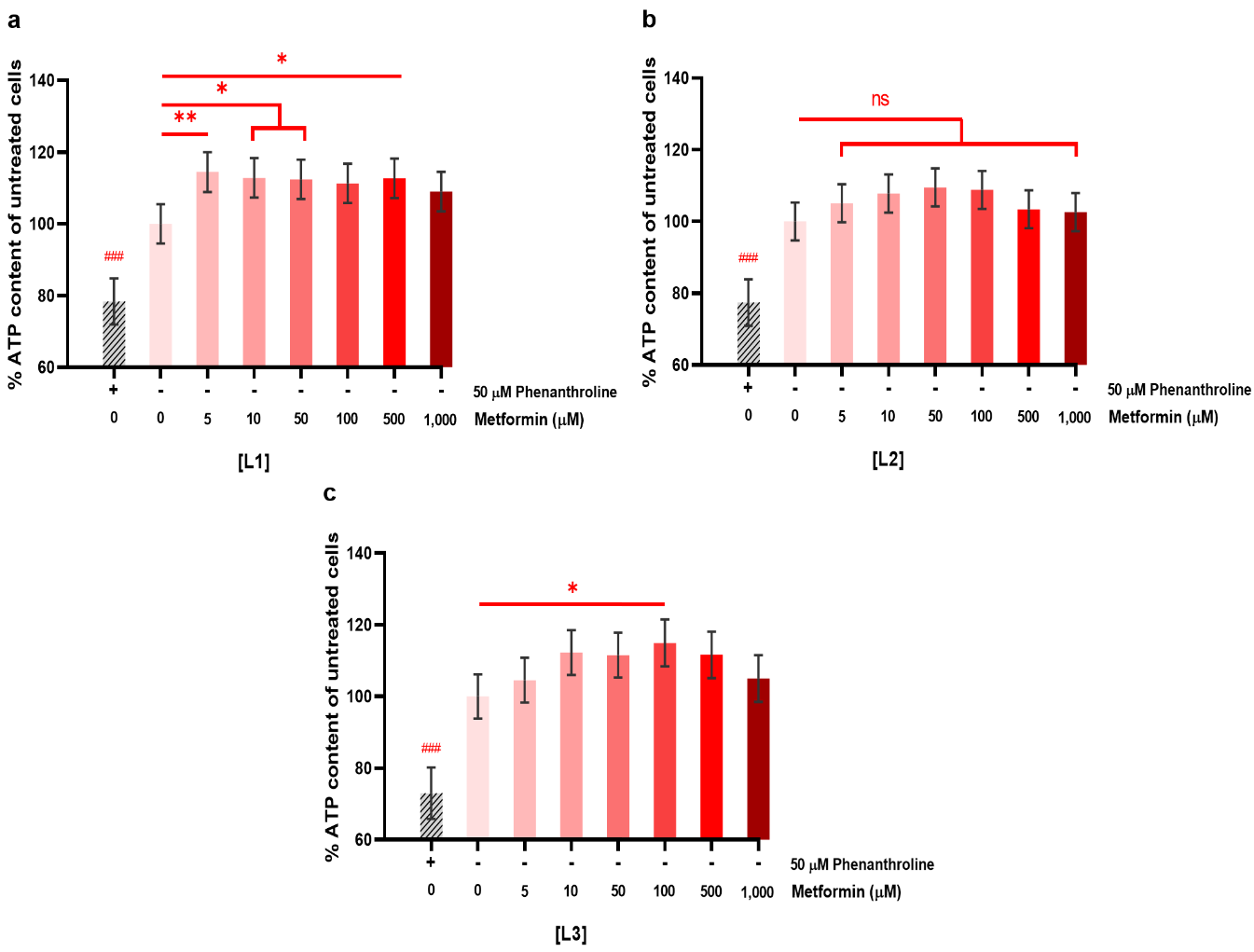


**A**

**C**

**B**

**Supplemental Figure S11. Metformin enhances ATP generation in specific LHON fibroblast samples.** ATP (%) after 24 h metformin vs untreated. Significant increases at (A) 5-50 and 500 µM (L1), (C) 100 µM (L3); trend in (B) L2. Phenanthroline (50 µM) lowers ATP. Design: 3-5 experiments/sample (total = 13); duplicate/triplicate wells. Bars: mean ± 95% CI. Statistics: ANOVA/Tukey; **p* < 0.05, ***p* < 0.01, ****p* < 0.001, ### phenanthroline vs untreated.

**B**

**A**


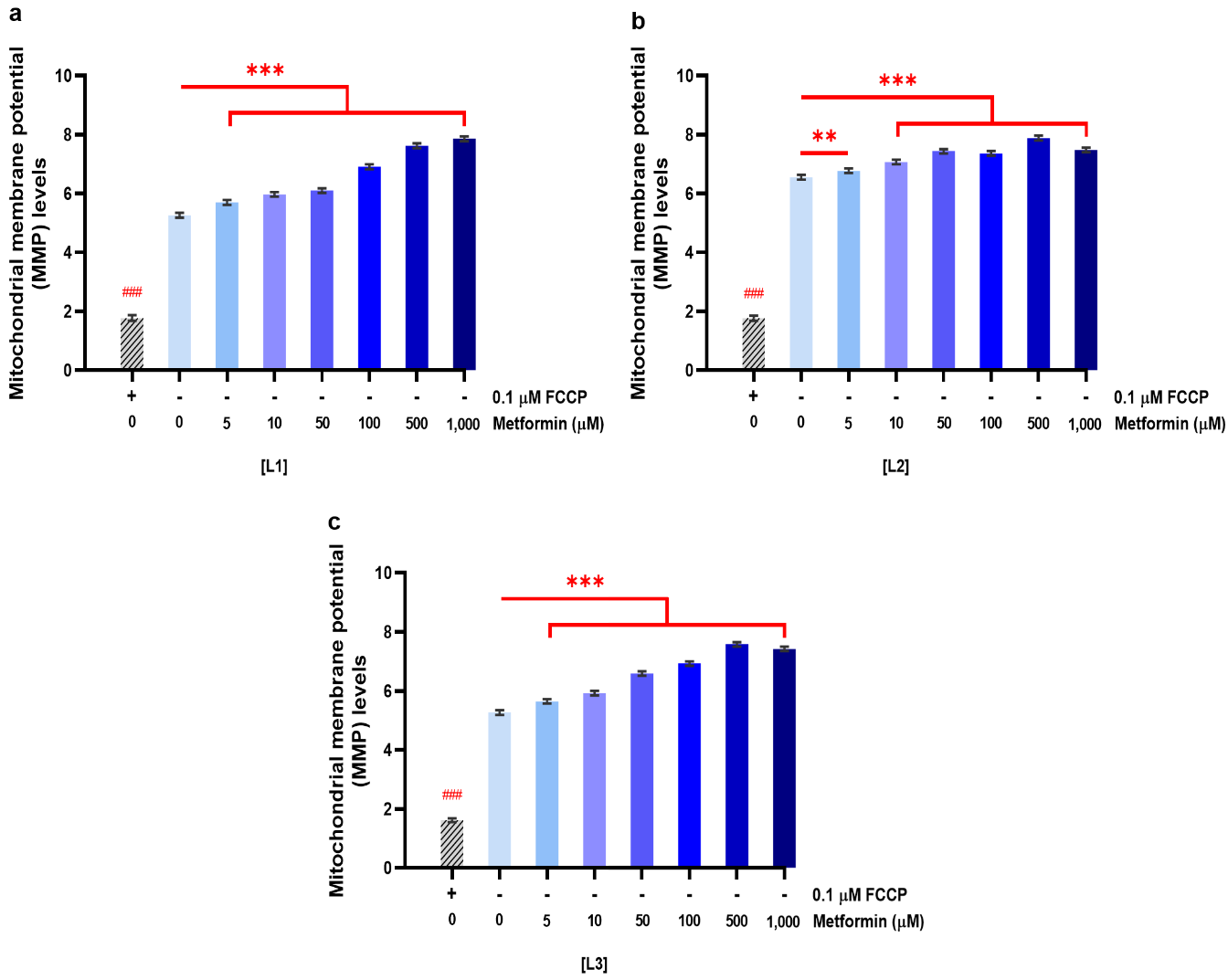


**C**

**Supplemental Figure S12. Metformin increases mitochondrial membrane potential (MMP) in LHON fibroblasts.** MMP rises after 24 h metformin at 5-1,000 µM in (A) L1, (B) L2, (C) L3. FCCP (0.1 µM) decreases MMP, serving as a control. Design: 3 experiments/sample; duplicate/triplicate wells; 65-75 fields/well, 15-35 cells/field. Bars: mean ± 95% CI. Statistics: ANOVA/Tukey; **p* < 0.05, ***p* < 0.01, ****p* < 0.001, ### FCCP vs untreated.

**A**

**
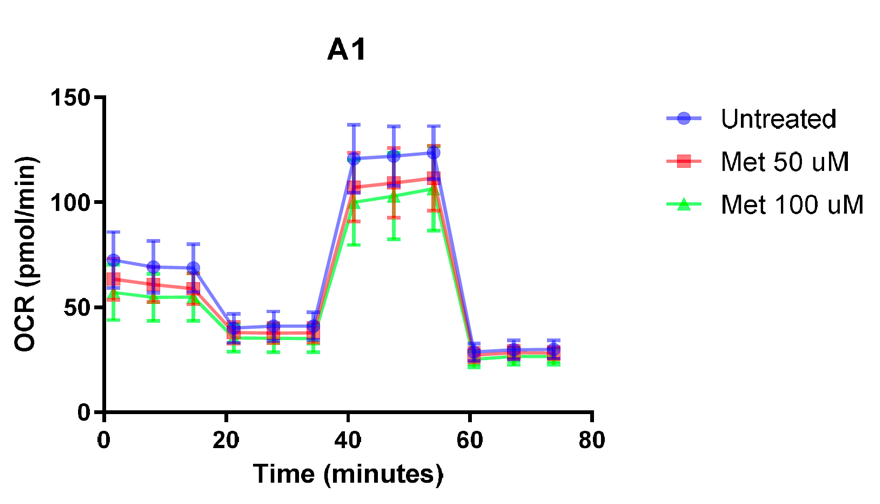
**

**L1**

**B**

**
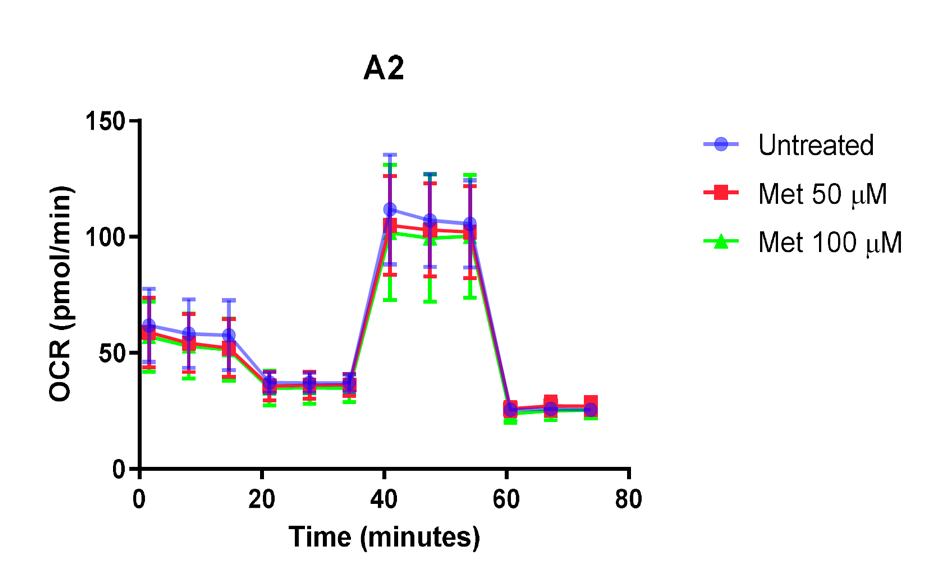
**

**L2**

**C**


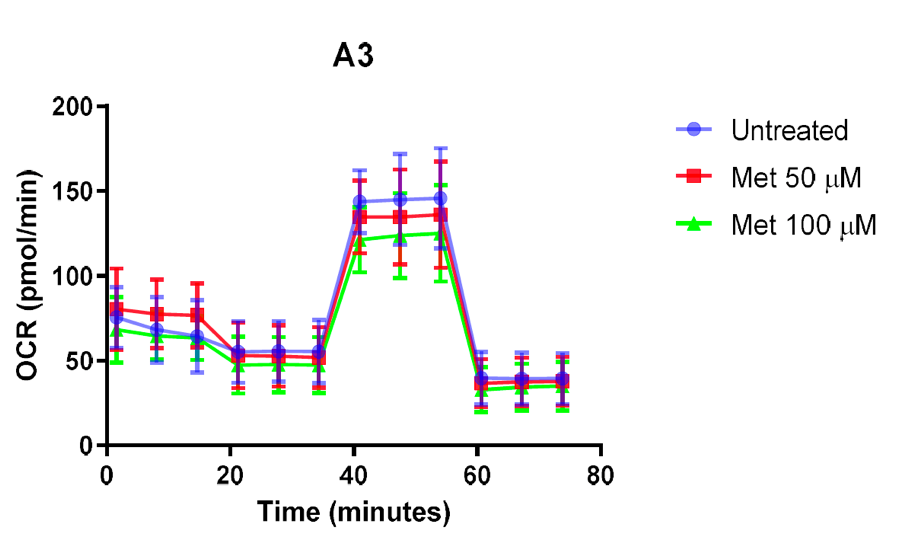


**L3**

**Supplemental Figure S13. Seahorse oxygen consumption rate (OCR) traces of LHON fibroblasts.** (A) L1, (B) L2, (C) L3 treated with metformin (50, 100 µM, 24 h). Oligomycin (5 µM), FCCP 2 (µM), antimycin A/rotenone (0.5 µM) were sequentially injected. Design: two wells/dose/experiment; 3 experiments/sample. Curves: mean ± 95% CI. Statistics: ANOVA/Tukey; ns = not significant.


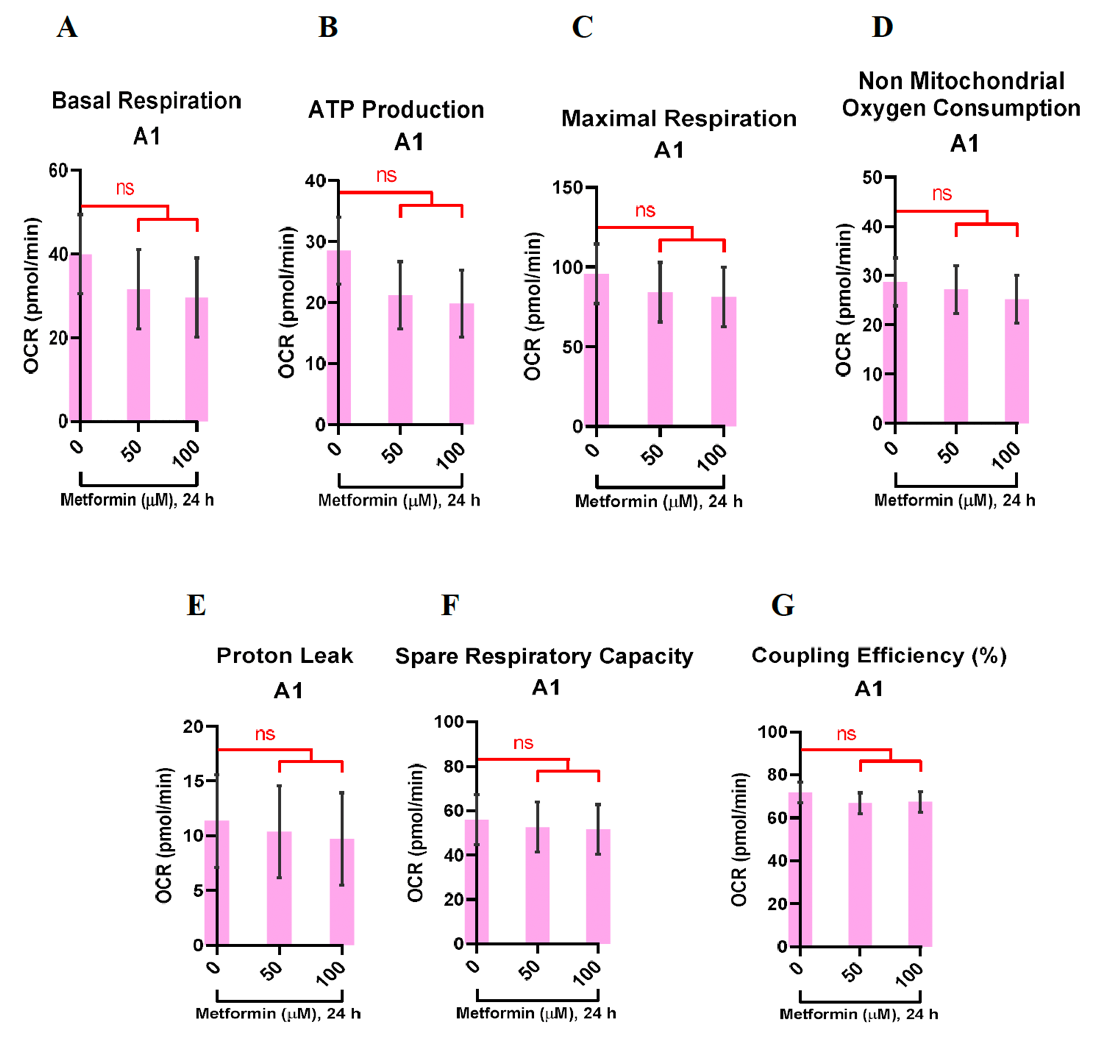


**L1**

**L1**

**L1**

**L1**

**L1**

**L1**

**L1**

**Supplemental Figure S14.** **Respiratory parameters in L1 fibroblasts treated with metformin.** Basal, ATP-linked, maximal, non-mitochondrial O₂ consumption, proton leak, spare capacity, coupling efficiency derived from OCR with inhibitors above. Design: duplicate wells/experiment; 3 experiments. Bars: mean ± 95% CI. Statistics: ANOVA/Tukey; ns = not significant.


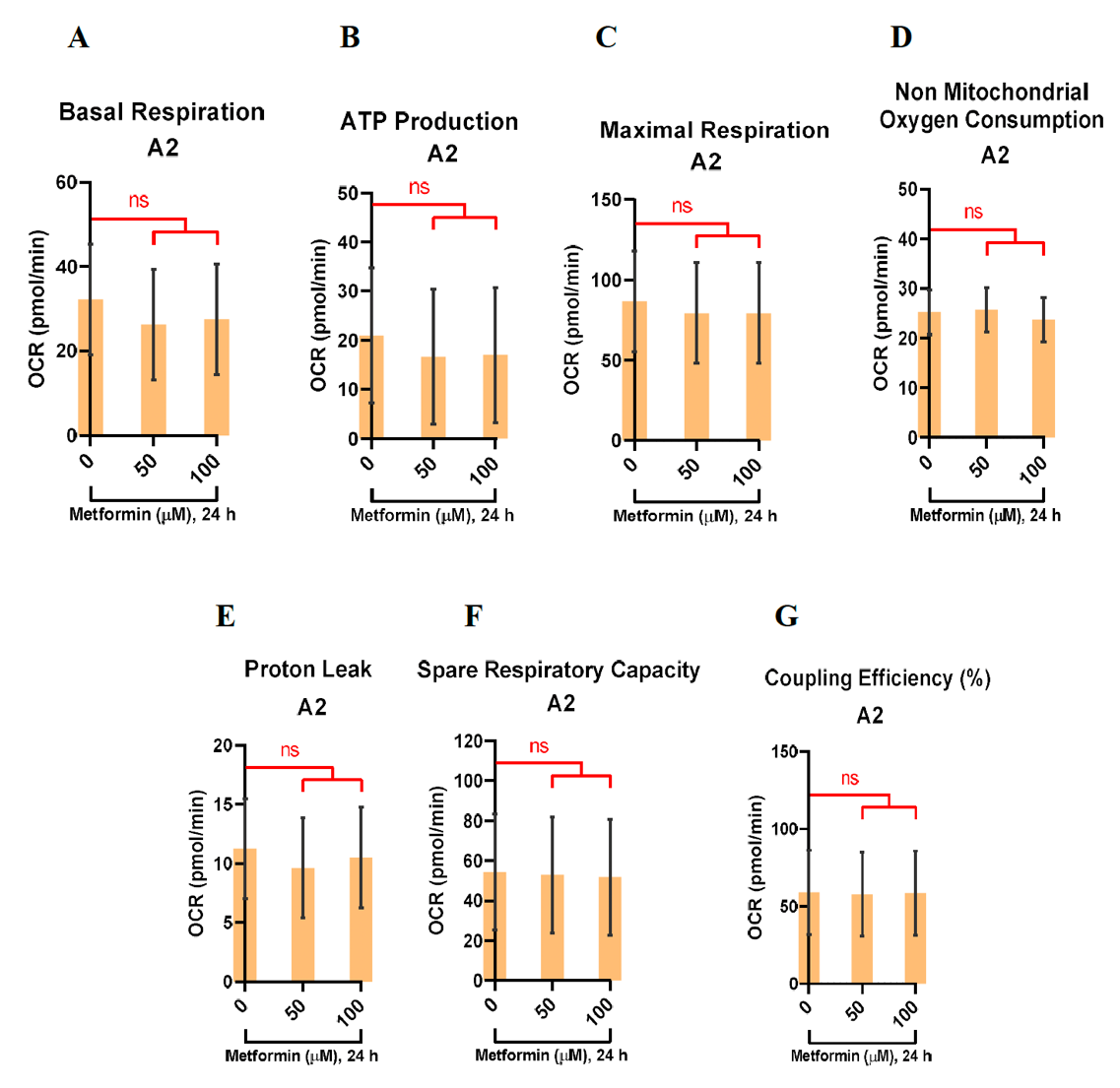


**L2**

**L2**

**L2**

**L2**

**L2**

**L2**

**L2**

**Supplemental Figure S15.** **Respiratory parameters in L2 fibroblast treated with metformin.** Design and statistics as in S21.


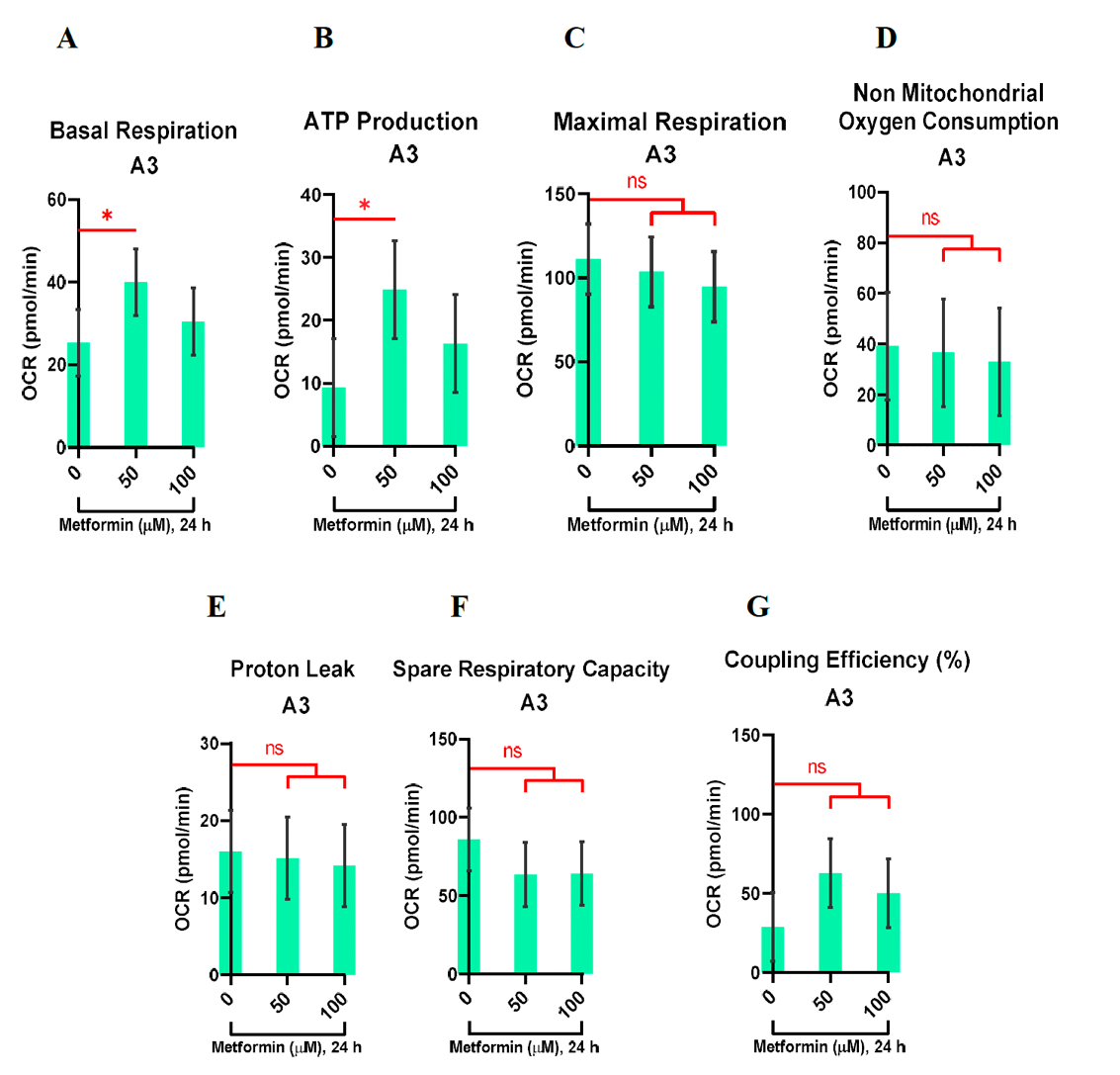


**L3**

**L3**

**L3**

**L3**

**L3**

**L3**

**L3**

**Supplemental Figure S16.** **Respiratory parameters in L3 fibroblasts treated with metformin.** Design and statistics as in S21.


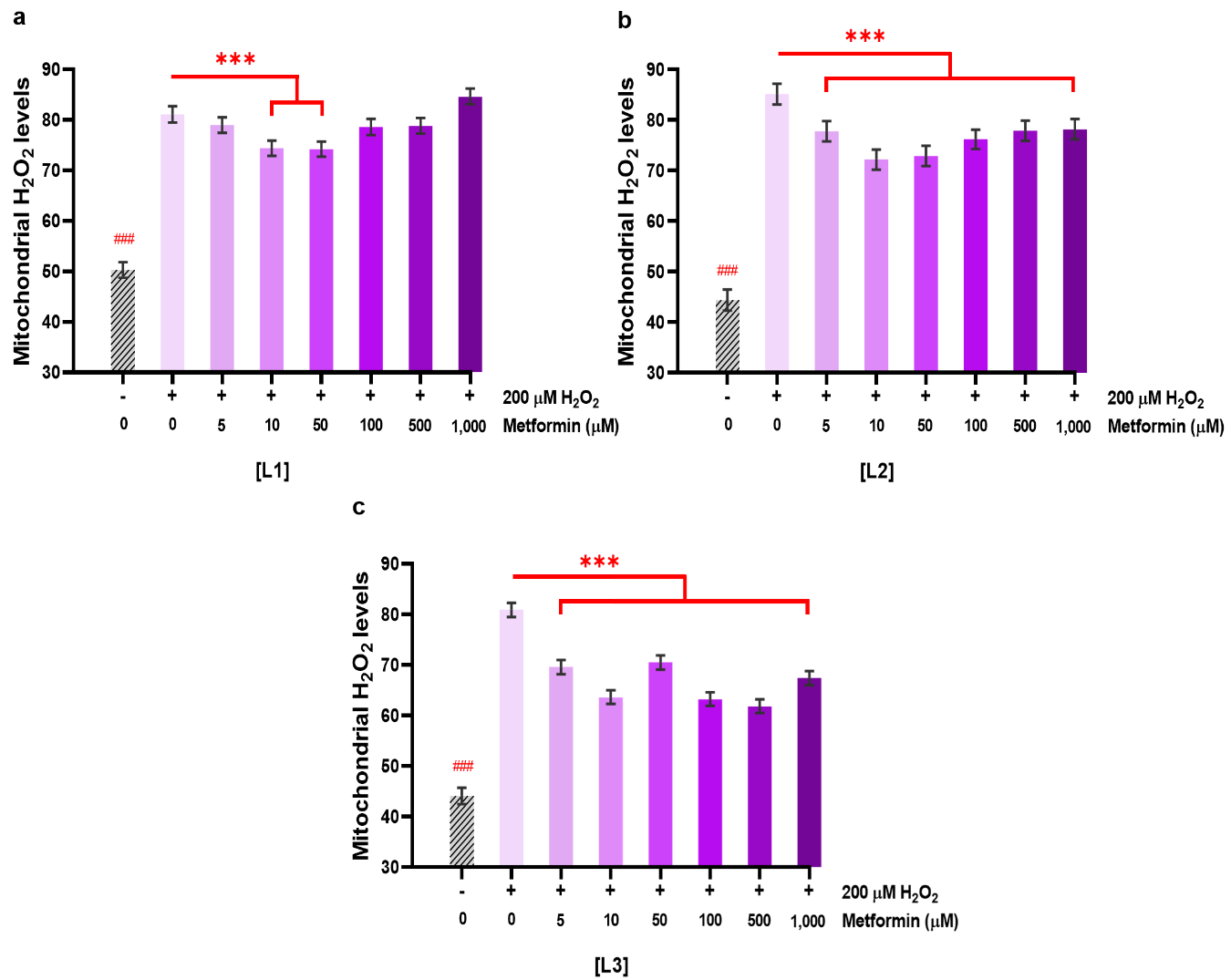


**A**

**C**

**B**

**Supplemental Figure S17.** **Metformin alleviates H₂O₂-induced mitochondrial oxidative stress in LHON fibroblasts.** After metformin (24 h) with H₂O₂-induced stress (200 µM, 1 h), mitochondrial H₂O₂ per cell decreases at (A) 10-50 µM (L1), (B) 5-1,000 µM (L2), (C) 5-1,000 µM (L3) vs H₂O₂ alone. Untreated controls show low H₂O₂. Design: 3 experiments/sample; duplicate wells; 50-60 fields/well, 15-35 cells/field. Bars: mean ± 95% CI. Statistics: ANOVA/Tukey; **p* < 0.05, ***p* < 0.01, ****p* < 0.001, ### untreated vs H₂O₂.


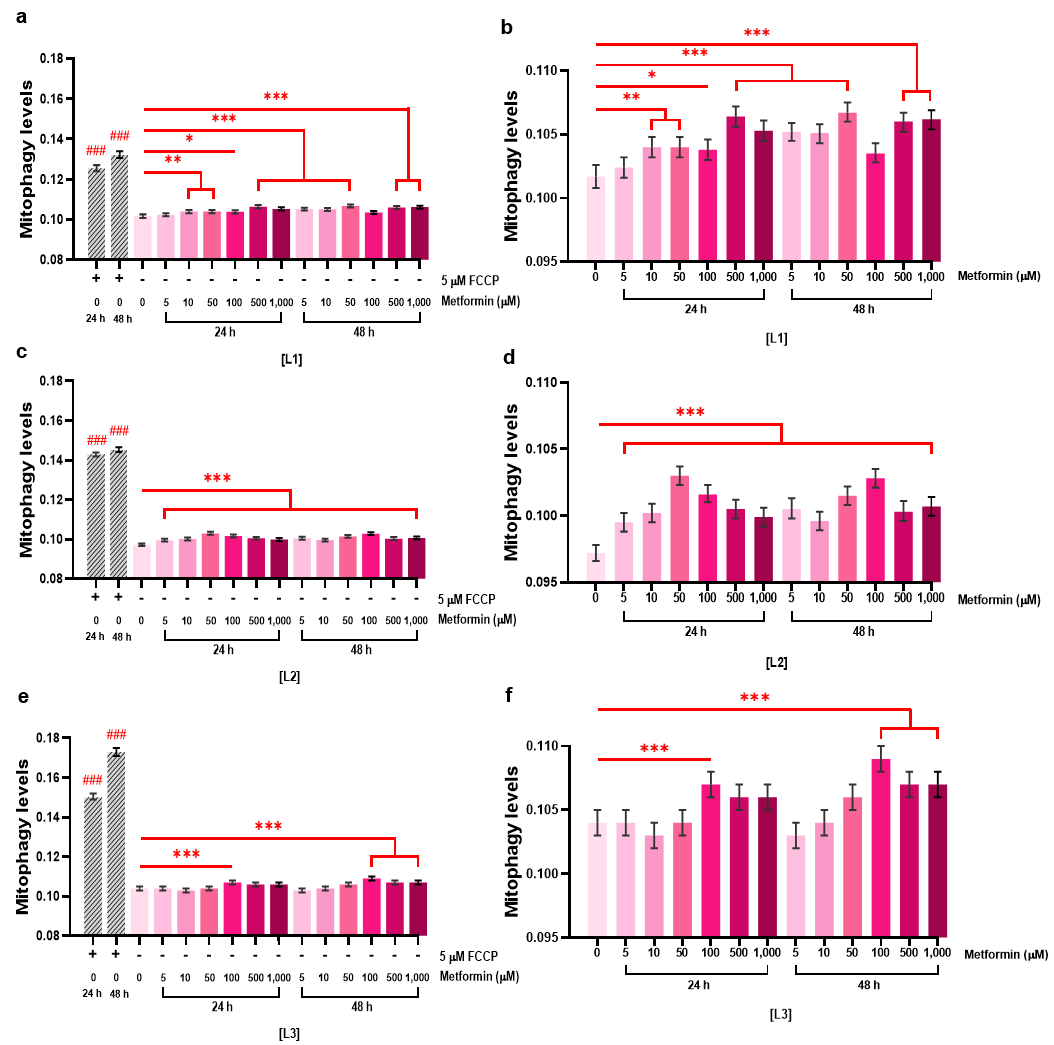


**E**

**C**

**A**

**F**

**B**

**D**

**Supplemental Figure S18.** **Metformin enhances mitophagy in LHON fibroblasts.** Left: mito-lyso colocalization normalized to mitochondria increases with metformin (5-1,000 µM; 24 and 48 h). FCCP (5 µM) as positive control. Right: Y-axis was rescaled excluding FCCP for clarity. Design: ≥3 experiments/sample; duplicate wells; 35-50 fields/well (160-180 cells/field), totaling 11,200-18,000 cells/condition/experiment. Bars: mean ± 95% CI. Statistics: ANOVA/Tukey; **p* < 0.05, ***p* < 0.01, ****p* < 0.001, ### FCCP vs untreated.

**
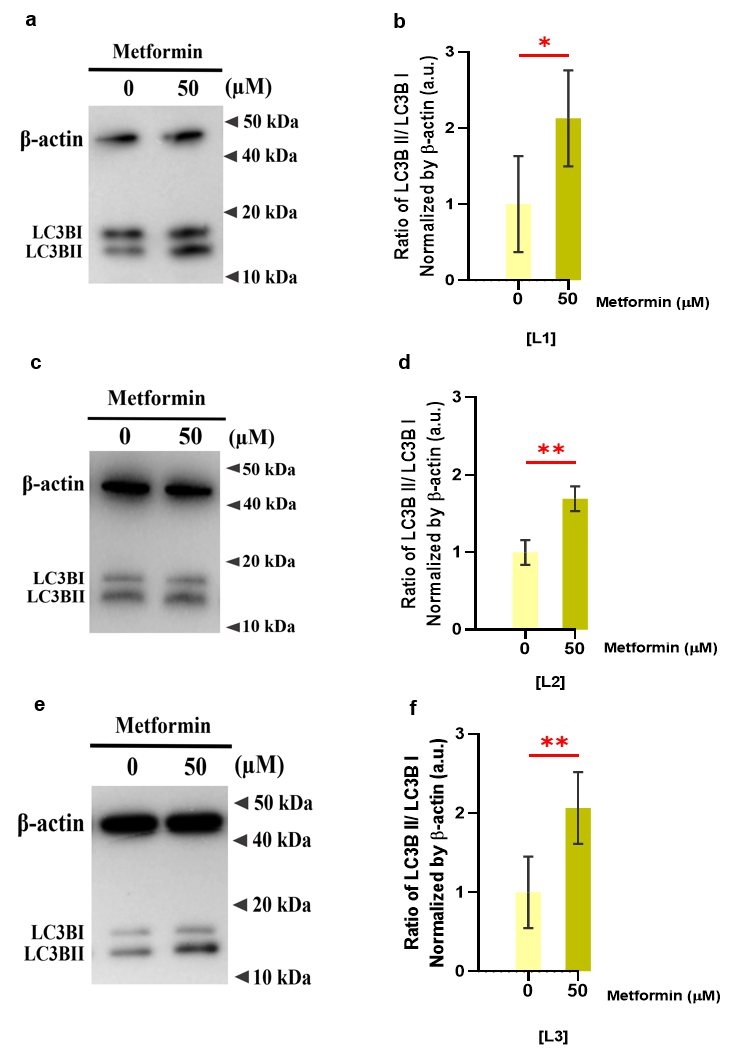
**

**E**

**C**

**A**

**F**

**D**

**B**

**Supplemental Figure S19. Metformin upregulates autophagic proteins in LHON fibroblasts.** Left: immunoblots (LC3B-II, LC3B-I, β-actin) after 50 µM, 24 h. Right: LC3B-II/LC3B-I normalized to β-actin; untreated = 1. Design: ≥3 experiments. Bars: mean ± 95% CI. Statistics: two-sided Student’s t-test (metformin vs. untreated); **p* < 0.05, ***p* < 0.01, ****p* < 0.001.

**B**

**A**


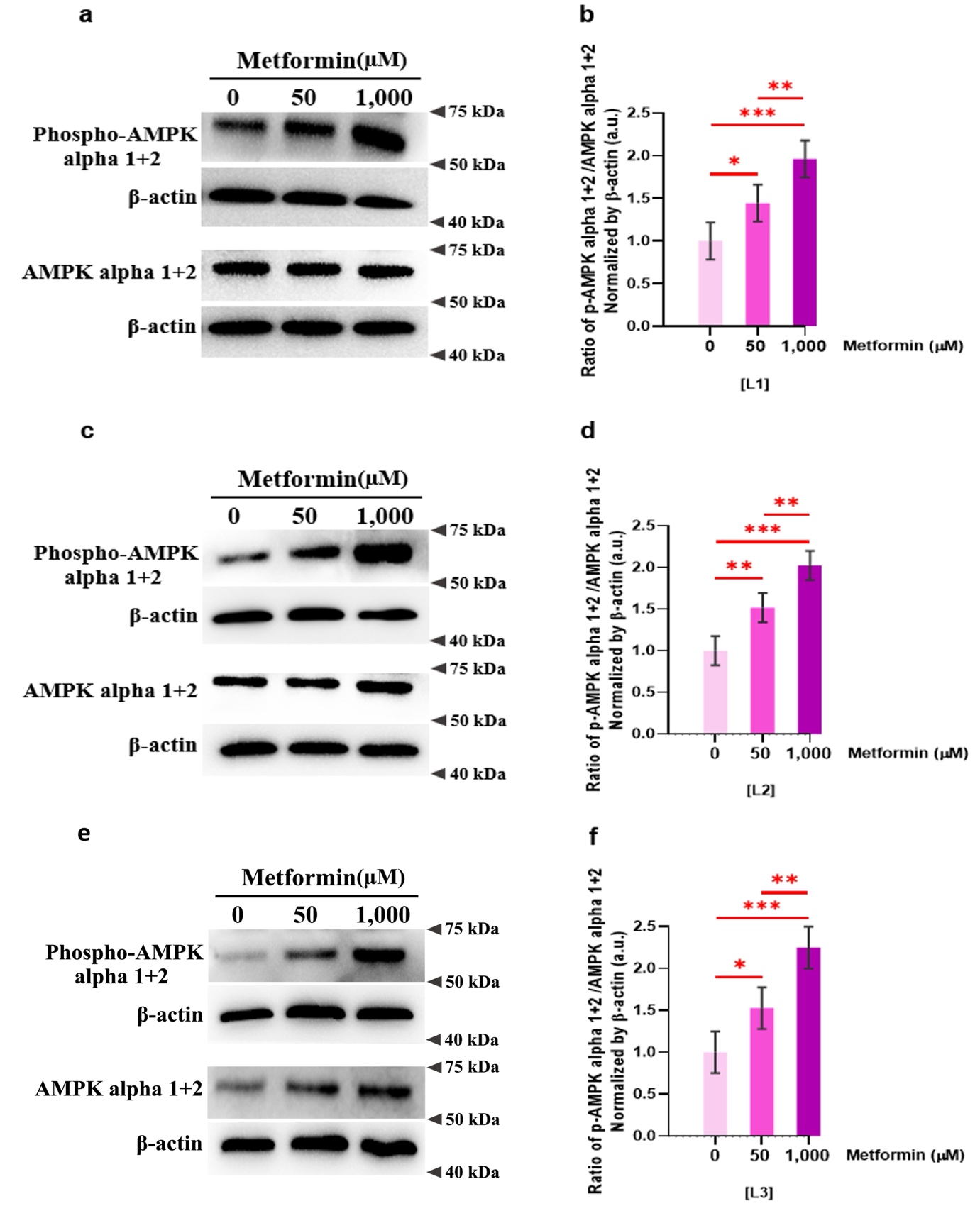


**D**

**E**

**F**

**C**

**Supplemental Figure S20. Metformin increases AMPK activation in LHON fibroblasts.** Left: Representative immunoblots showing p-AMPKα1 (Thr183)/α2 (Thr172); total AMPKα1/α2; β-actin after 50 and 1,000 µM, 24 h. Right: p-AMPK/total AMPK normalized to β-actin; untreated = 1. Design: ≥3 experiments/sample. Bars: mean ± 95% CI. Statistics: one-way ANOVA/Tukey; **p* < 0.05, ***p* < 0.01, ****p* < 0.001.

**Supplemental Figure S21.** **pAMPKβ1 levels are lower in LHON fibroblasts compared with healthy controls.** Quantification of AMPKβ1 phosphorylation (Ser182) fluorescence intensity normalized to DAPI nuclear staining in individual healthy control and LHON fibroblasts.

**A B**

**** ****

**C**

**
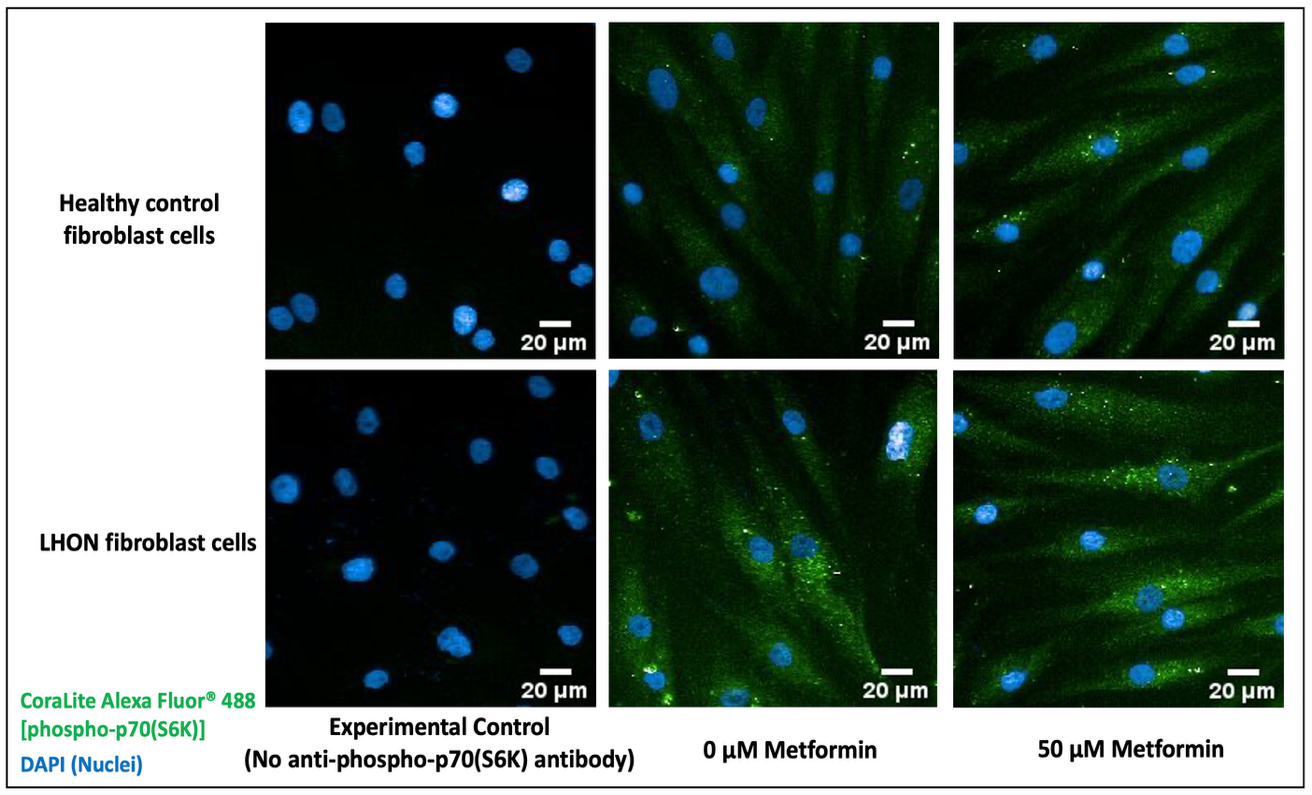
**

**Supplemental Figure S22.** **Metformin increases phospho-p70(S6K) (Thr389) in healthy control fibroblasts but not in LHON fibroblasts.** (A) Quantification of phospho-p70(S6K) fluorescence intensity in healthy controls and (B) LHON fibroblasts under metformin treatment (50 µM, 24 h) versus untreated. (C) Representative images of phospho-p70(S6K) (green) in metformin-treated and untreated fibroblasts; nuclei counterstained with DAPI. Imaging: 20x objective; scale bar, 20 µm. Design: 25-45 fields/well, 2,500-9,000 cells/condition, duplicate. n = 3/group. Statistics: Student’s t-test; **p* < 0.05, ***p* < 0.01, ****p* < 0.001.


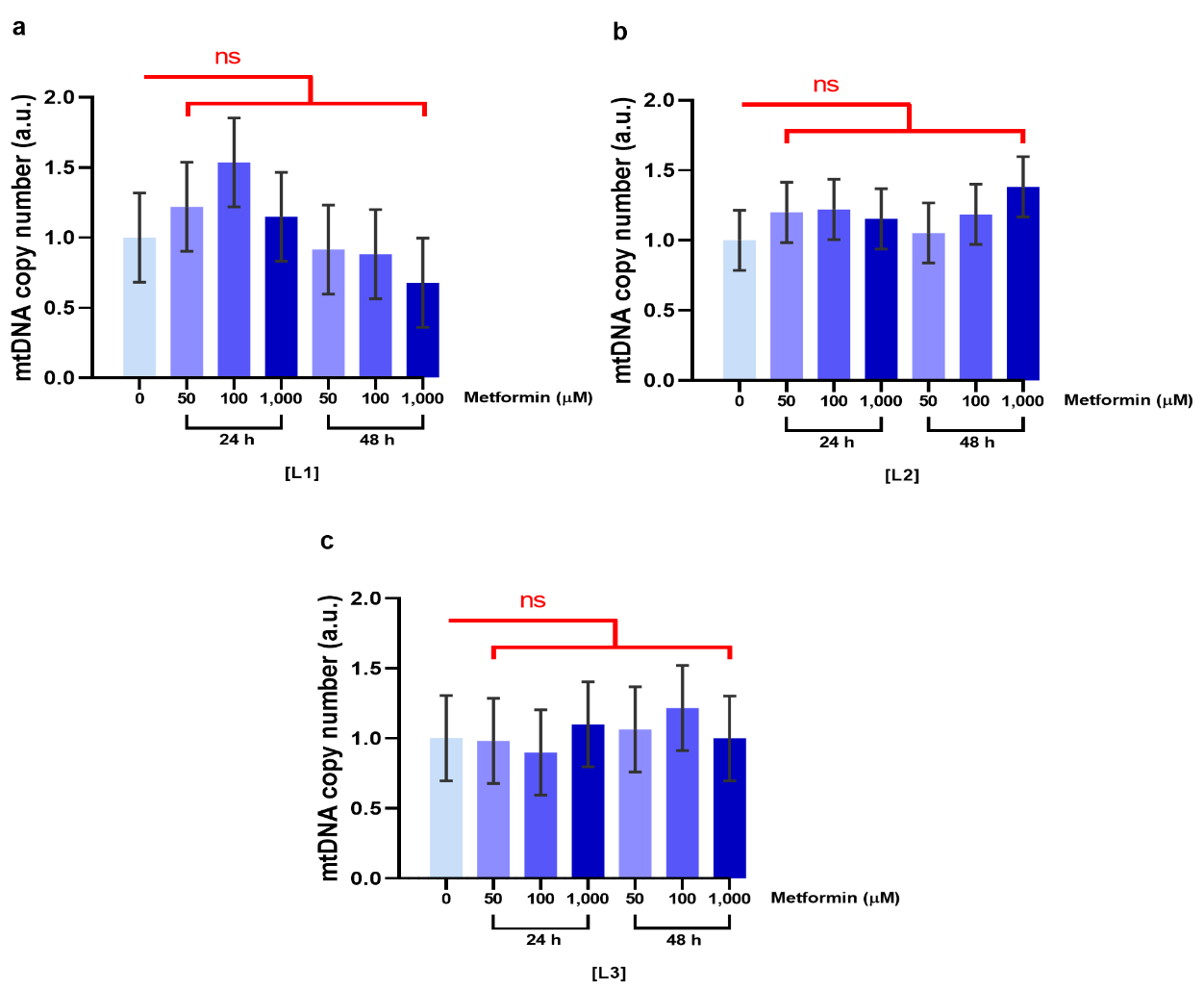


**C**

**B**

**A**

**Supplemental Figure S23.** **Metformin does not alter mitochondrial mass in LHON fibroblasts.** mtDNA copy number (*MT-TL1*/*ZHX2*) after 50, 100, 1,000 µM metformin for 24 and 48 h in (A) L1, (B) L2, (C) L3. Design: duplicate wells; ≥3 experiments/sample. Bars: mean ± 95% CI. Statistics: one-way ANOVA/Tukey; ns = not significant.

**A B**

**C**

**Supplemental Figure S24.**  **Metformin induces PGC-1α nuclear translocation in specific LHON fibroblast samples.** (A) L1 fibroblasts showed no significant change in PGC-1α nuclear localization after 24 h of treatment with 50 µM metformin. (B-C) L2 and L4 fibroblasts displayed significantly increased PGC-1α nuclear translocation under the same treatment conditions. Analysis was performed with CellProfiler. Design: 25-45 fields/well (50-100 cells/field); 2,500-9,000 cells/condition in duplicate. Bar graphs: mean ± 95% CI. Statistics: one-way ANOVA/Tukey; **p* < 0.05, ***p* < 0.01, ****p* < 0.001.


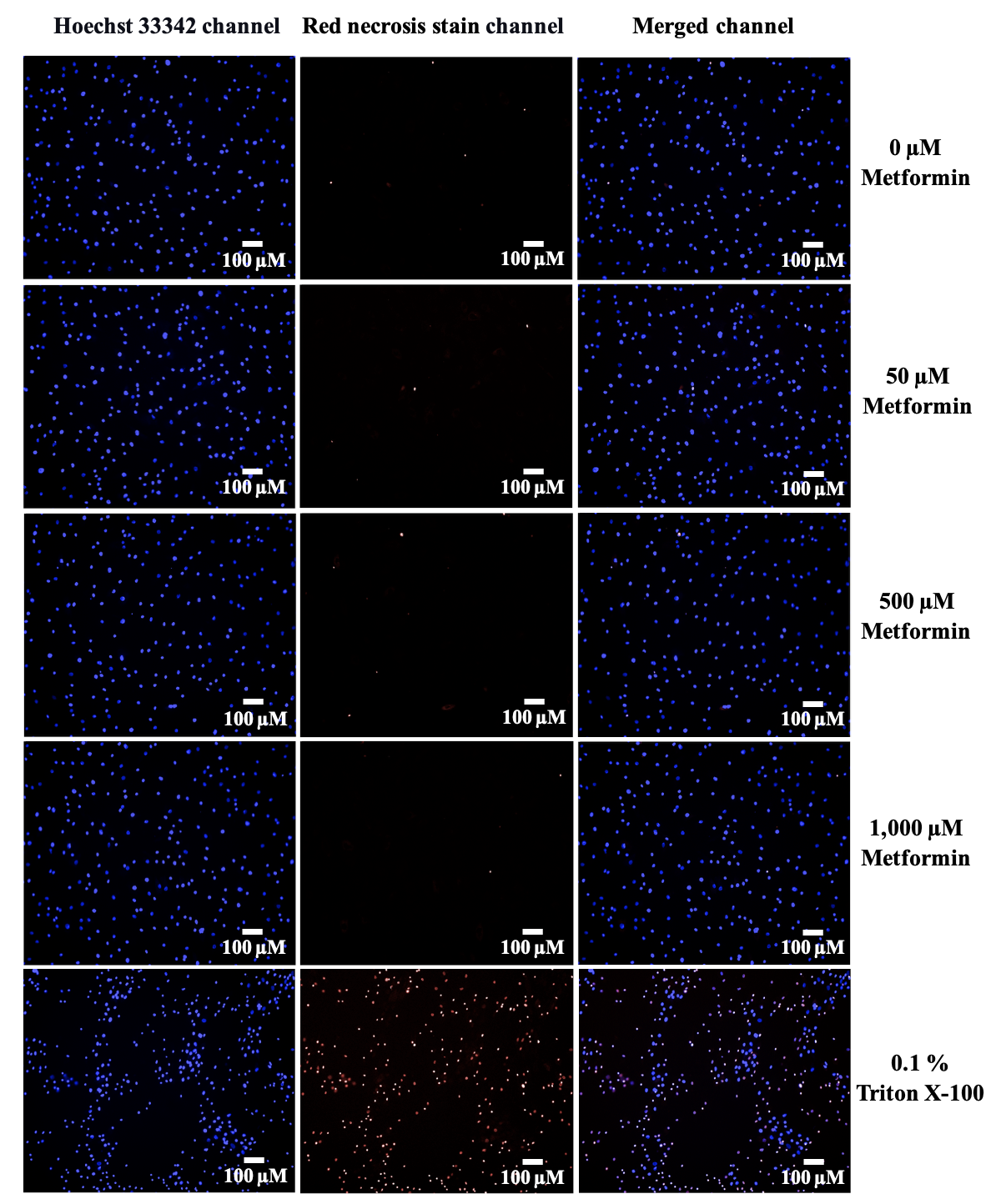


**Supplemental Figure S25.** **Cytotoxicity imaging in fibroblasts treated with metformin.** Representative images after 48 h metformin (50, 500, 1,000 µM) and 0.1% Triton X-100 (positive control, 5 s). Hoechst (total nuclei, blue); necrosis dye (red). Imaging: Operetta CLS™, 10× high-NA air objective. Scale bars, 100 µm.

**A B**


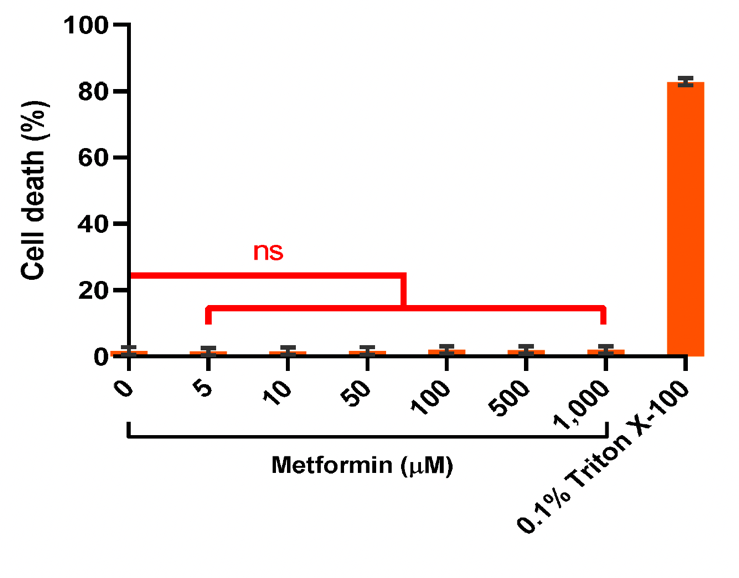

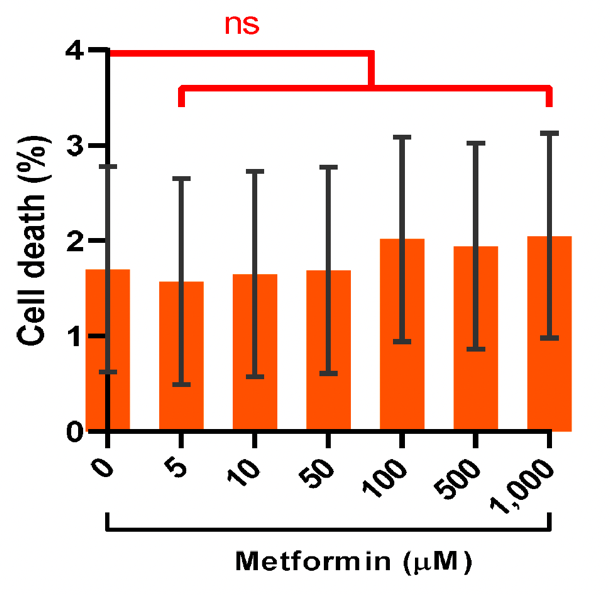


**Supplemental Figure S26. Quantitative analysis of cytotoxicity in LHON fibroblasts following metformin treatment.** (A) Percentage cell death calculated as red-overlapping-blue/total blue nuclei after metformin (5-1,000 µM, 48 h) or 0.1% Triton X-100 (5 s). (B) Y-axis rescaled; Triton control omitted for clarity. Data from 12 experiments using four LHON fibroblast samples (L1-L4). Design: duplicate wells; 15 fields/well (~300 cells/field), ≈9,000 cells/condition/experiment. Bars represent mean ± 95% CI. Statistics: two-way ANOVA with Tukey’s test; ns = not significant.

**References**

1. Panusatid, C., Thangsiriskul, N. & Peerapittayamongkol, C. (2022) Methods for mitochondrial health assessment by High Content Imaging System, *MethodsX*, 101685.

2. Redmann, M., Benavides, G. A., Wani, W. Y., Berryhill, T. F., Ouyang, X., Johnson, M. S., Ravi, S., Mitra, K., Barnes, S., Darley-Usmar, V. M. & Zhang, J. (2018) Methods for assessing mitochondrial quality control mechanisms and cellular consequences in cell culture, *Redox biology.* **17**, 59-69.
